# Real-Time Multistep Asymmetrical Disassembly of Nucleosomes and Chromatosomes Visualized by High-Speed Atomic Force Microscopy

**DOI:** 10.1021/acscentsci.3c00735

**Published:** 2023-12-22

**Authors:** Bibiana Onoa, César Díaz-Celis, Cristhian Cañari-Chumpitaz, Antony Lee, Carlos Bustamante

**Affiliations:** † Jason L. Choy Laboratory of Single-Molecule Biophysics, University of California, Berkeley, California 94720, United States; ‡ Howard Hughes Medical Institute, University of California, Berkeley, California 94720, United States; § California Institute for Quantitative Biosciences, QB3, University of California, Berkeley, California 94720, United States; ∥ Laboratoire Photonique Numérique et Nanosciences, LP2N UMR 5298, Université de Bordeaux, Institut d’Optique, CNRS, F-33400 Talence, France; ⊥ Kavli Energy Nanoscience Institute, University of California, Berkeley, California 94720, United States

## Abstract

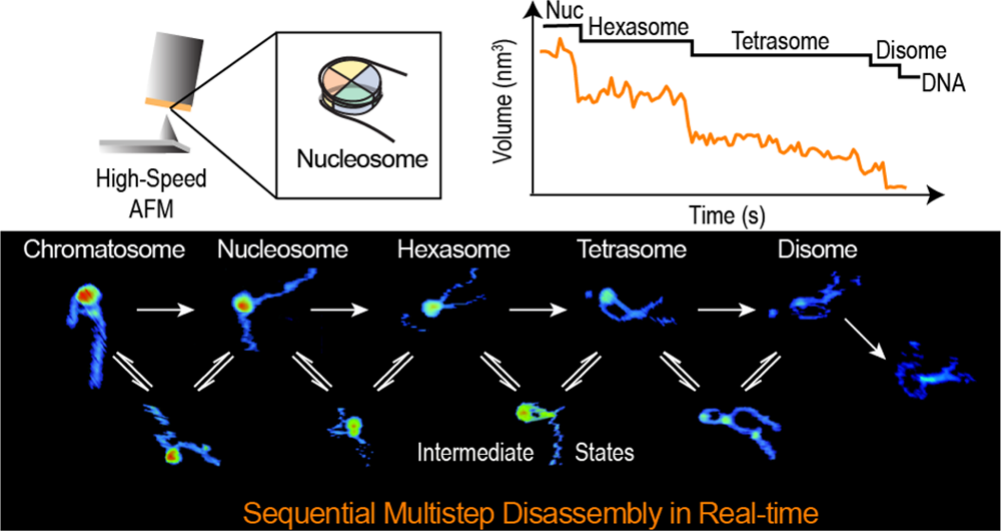

During replication, expression, and repair of the eukaryotic genome, cellular machinery must access the DNA wrapped around histone proteins forming nucleosomes. These octameric protein·DNA complexes are modular, dynamic, and flexible and unwrap or disassemble either spontaneously or by the action of molecular motors. Thus, the mechanism of formation and regulation of subnucleosomal intermediates has gained attention genome-wide because it controls DNA accessibility. Here, we imaged nucleosomes and their more compacted structure with the linker histone H1 (chromatosomes) using high-speed atomic force microscopy to visualize simultaneously the changes in the DNA and the histone core during their disassembly when deposited on mica. Furthermore, we trained a neural network and developed an automatic algorithm to track molecular structural changes in real time. Our results show that nucleosome disassembly is a sequential process involving asymmetrical stepwise dimer ejection events. The presence of H1 restricts DNA unwrapping, significantly increases the nucleosomal lifetime, and affects the pathway in which heterodimer asymmetrical dissociation occurs. We observe that tetrasomes are resilient to disassembly and that the tetramer core (H3·H4)_2_ can diffuse along the nucleosome positioning sequence. Tetrasome mobility might be critical to the proper assembly of nucleosomes and can be relevant during nucleosomal transcription, as tetrasomes survive RNA polymerase passage. These findings are relevant to understanding nucleosome intrinsic dynamics and their modification by DNA-processing enzymes.

## Abstract 2

Our study provides strong structural evidence of how eukaryotic DNA is accessed, capturing in real time the multistep disassembly of nucleosomes, chromatosomes, and subnucleosomal particles by high-speed AFM.

## Introduction

The long genomes of eukaryotes are organized into chromatin to fit in the micrometer-sized nuclear space. Chromatin, mainly composed of DNA and histone proteins, is compacted in a way that protects the DNA while it enables its replication, transcription, and repair.^1^ The nucleosome is the structural unit of chromatin and is made up of ∼1.7 turns of DNA (147 bp) wrapped around a core of histones H2A, H2B, H3, and H4 assembled into a stable albeit dynamic and flexible octameric structure [(H3·H4)_2_·(H2A·H2B)_2_]. The resulting nucleoprotein complex constitutes the nucleosome core particle (NCP)^2,3^ which can be further compacted by binding linker histones (e.g., H1 or H5) to form chromatosomes. Nucleosomes are epigenetically modified to tightly regulate their assembly and disassembly as well as the internucleosomal interactions established to acquire higher-order 3D structures. The nucleosomal modular architecture confers plasticity to initiate DNA-templated processes in response to cellular signals.^1^

Nucleosomes and partially assembled nucleosomal structures (PANS) such as hexasomes, tetrasomes, and disomes are thought to play a pivotal role in the regulation of gene expression.^4,5^ Accordingly, great interest exists in understanding the mechanisms by which histone·histone and histone·DNA interactions are modified to expose key DNA sequences recognized by proteins processing the genome throughout the cell’s life cycle. Studies of nucleosome dynamics have revealed that subnucleosomal intermediates exist in the cell, presumably by the action of chromatin remodelers, polymerases, histone chaperones, etc.^1,4,5^ The picture emerging today is one in which nucleosomes likely exist as a mixture of highly dynamic interconverting structural states in transcriptionally active regions,^6−8^ whereas in repressive regions they are compacted by linker histones and other associated proteins.^1,8^

Some of the dynamics observed in vivo have also been observed in vitro. When nucleosomes are diluted to nanomolar concentrations, they spontaneously unwrap and, under some conditions, fully dissociate,^9−12^ giving rise to a repertoire of PANS,^13,14^ even when they are assembled using the synthetic and strong 601 nucleosome positioning sequence (NPS).^15−18^ Dynamic studies have been predominantly circumscribed to the use of techniques to separately monitor the unwrapping of DNA from the histone core or the dissociation of the histones in high-ionic-strength environments (time-resolved small-angle X-ray scattering (TR-SAXS), single-molecule Förster resonance energy transfer (sm-FRET), directional mechanical force (optical/magnetic tweezers),^15−23^ and nonphysiological temperatures (>70 °C).^24^ Those studies have established that nucleosomes disassemble sequentially, forming PANS, and that the unwrapping of DNA is not symmetric but rather starts from the rigid side of the NPS followed by the flexible one. Consequently, nucleosomes behave as polarized barriers for RNA polymerases, making transcription in one direction more efficient than in the other.^25^ While these in vitro experiments provide insight into the dynamics of nucleosome unwrapping and disassembly, they do not directly provide structural information. Molecular dynamics simulations of nucleosomes and PANS provide an atomistic description of their dynamics at high temporal resolution but are limited to submicrosecond-scale trajectories.^13^ Accordingly, a real-time method to visualize nucleosome disassembly and track DNA and NCP dynamics under physiological conditions is still missing.

Atomic force microscopy (AFM) has been used to investigate nucleosomes and chromatin structure both in air and in aqueous environments.^14,26−36^ A recent AFM study reported that nucleosome unwrapping occurs preferentially on the stiffer side of 601 NPS (53.7 ± 1.6% of the time).^28^ High-speed AFM (HS-AFM) allowed us to visualize and kinetically characterize the dynamics—unwrapping, looping, sliding, and histone dissociation—of nucleosomes as well as internucleosomal interactions.^29−32,34,35,37,38^ Here, we used HS-AFM to observe the disassembly in real time of individual nucleosomes, chromatosomes, and their PANS in a physiological environment. In particular, we aimed to monitor nucleosome dynamic dissociation by tracking the NCP volume changes over time to address the following questions: (1) What is the fate of the disassembled particles and the newly accessible DNA? (2) Is the disassembly pathway modified in chromatosomes when nucleosomal unwrapping is impaired by the binding of linker histone H1?^39^

Our results show that DNA unwrapping of nucleosomes as well as chromatosomes is asymmetric, and their disassembly is a multistep sequential process. We observed that DNA unwrapping is needed for nucleosomal dimer dissociation. By tracking the evolution of the histone core volume, we can distinguish the dynamics, rates, and pathways of disassembly of nucleosomes, hexasomes, and tetrasomes. For example, tetrasomes exhibit a longer lifespan than hexasomes or nucleosomes. The longer survival time of chromatosomes compared to that of nucleosomes indicates that the stabilization of DNA linkers by H1 is indeed critical to preserving their integrity. We demonstrated that regardless of the origin of tetrasomes, either residual after disassembly or purified as de novo particles, they diffuse around the dyad region of the 601 NPS; in de novo tetrasomes, there is a weak preference for the flexible over its rigid side. This feature might have relevance during assembly since the precise location of the tetramer might determine the structural fate and stability of nucleosomes.

## Results and Discussion

### Dynamic Disassembly of Nucleosomes and Subnucleosomal Particles in Real Time

To determine and visualize the spontaneous disassembly mechanism of nucleosomes deposited on mica, we reconstitute recombinant human nucleosomes on the 601 NPS flanked by DNA arms of 200 bp and 100 bp on the flexible and rigid sides, respectively. Nucleosomes were further purified and concentrated using polyacrylamide electrophoresis, a process outlined in the Methods, Supporting Information (SI), and Figure SI 1A. The concentrated sample (∼1.4 μM) was diluted ∼900-fold prior to deposition. As stated above, it has been established that the stability of the nucleosome cores is directly proportional to their concentration, even within the context of nucleosome arrays.^10,12^ We deposited the nucleosomes onto freshly cleaved and nonfunctionalized mica at concentrations of between 1.5 and 3.5 nM in magnesium-free buffer A (15 mM MOPS, pH 7.0, 80 mM KCl, 20 mM NaCl, 0.5 mM EGTA, 2 mM EDTA, 0.5 mM spermidine, 0.2 mM spermine, 5 mM Na(C_3_H_7_COO), 1 mM DTT) previously used to extract and preserve chromosomes from *Drosophila* embryos and nuclei from rat liver.^40,41^ Samples were imaged in 10-fold-diluted buffer A at one or two frames per second (fps). We focus on individual, intact nucleosomes to accurately discern their DNA arms and track the behavior of single NCPs. Importantly, prior to selecting nucleosomes for extended scanning, it was common to encounter a mixture of nucleosomes, PANS, and bare DNA on the surface (Figure SI 2A), consistent with our electrophoretic analysis (Supporting Information, Figure SI 1A). The relative amount of PANS observed by AFM could also be attributed to the substantial dilution required to observe individual nucleosomes.^10^ It is probable that a significant fraction of nucleosomes undergoes structural modifications before adsorption onto the surface, leading to the formation of diverse species and/or metastable configurations. All of the nucleosomes observed in this study underwent some level of disassembly.

**Figure 1:**
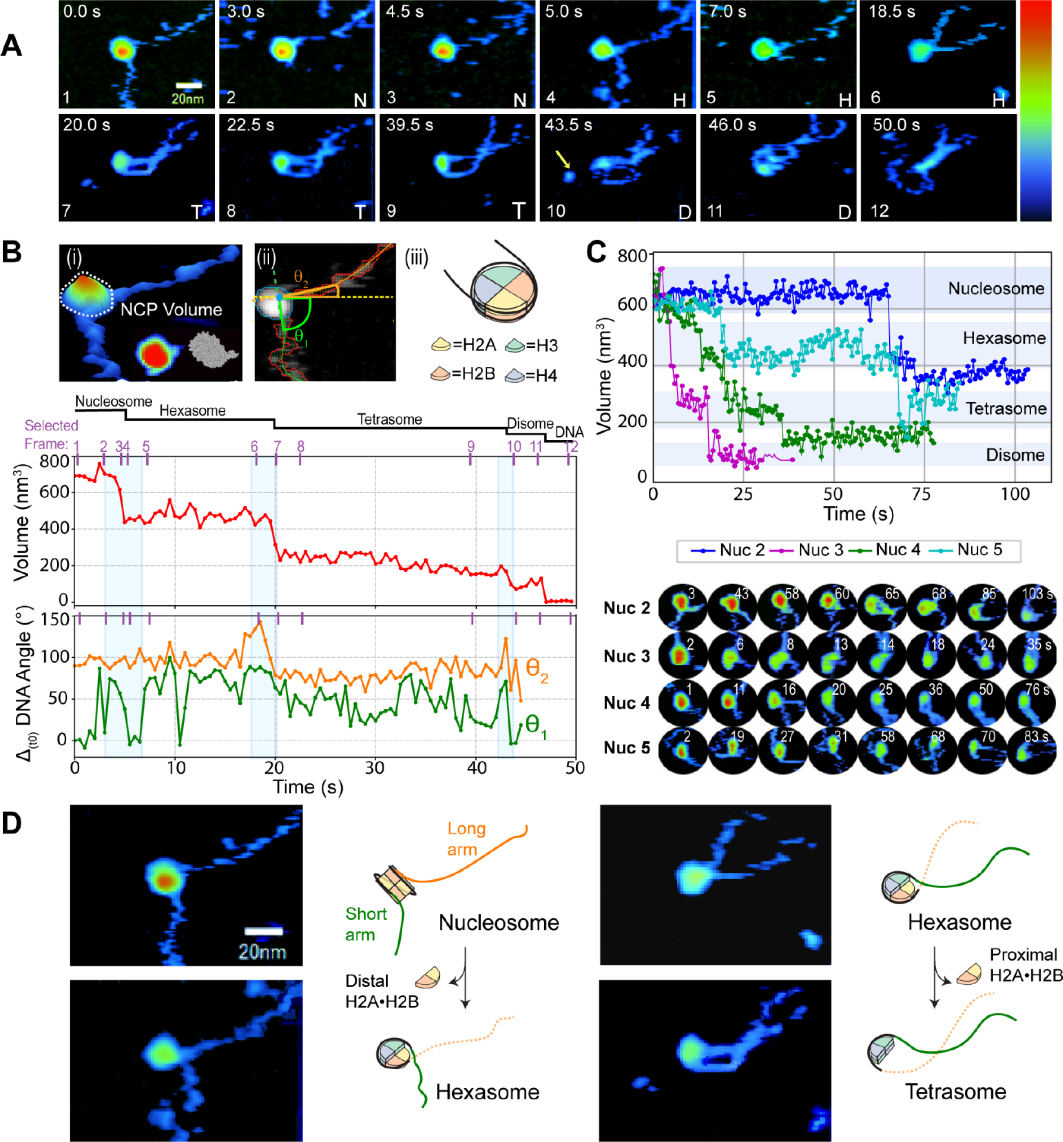
Dynamics of nucleosome disassembly. (A) HS-AFM time lapse of the morphological evolution of a nucleosome disassembly observed in buffer A at 2 fps (Movie SI 2). The yellow arrow indicates a histone-ejection event. Z-color-map from 0 to 6 nm. N = nucleosome, H = hexasome, T = tetrasome, and D = disome. (B) (i) 3D rendering of the nucleosome in (A) illustrating the NCP volume inside a white dotted circle. (Inset) Comparison of the experimental AFM NCP in (A) and its equivalent atomic surface map (PDB 5NL0 in BioAFMviewer^42^). (ii) Example of automatic segmentation in which the pixels used to compute NCP’s volume are enclosed by a blue line and the DNA arms are identified by red areas. Tracing of the short arm (flanking the 601 NPS rigid arm) and the long arm (flanking the 601 NPS flexible arm) are displayed as green and orange lines, respectively. Entry (θ_2_) or exit (θ_1_) angles were measured with respect to the *X* axis (dotted yellow line) traced from the intersect of the projection of the DNA traces inside the NCP (blue dot). (iii) Cartoon of a nucleosome depicting its structural components. Time evolution of the histone core volume (middle panel) and DNA arms angles relative to the entry and exit sites (bottom panel) of the nucleosome shown in (A). Angular fluctuations are relative to the value at time zero (Δ_(t0)_), with the trace corresponding to θ_2_ offset upward for clarity (bottom panel). Purple ticks and numbers in the plots show the selected time point displayed in the corresponding time-lapse sequence in (A). Blue-shaded rectangles simultaneously indicate DNA angle and NCP volume changes due to a dimer ejection event. (C) Examples of four additional nucleosome volume trajectories as a function of time (top panel) and their representative time-lapse AFM images (bottom panel). Shadowed regions indicate the range of volume observed for different PANS. (D) Selected AFM micrographs from the time-lapse sequence in A and corresponding cartoons depicting the molecular structural changes (histone core morphology and DNA arms’ displacement) upon each H2A·H2B heterodimer dissociation event.

Nucleosomes exhibit a wide range of mobility and morphological configurations under our experimental conditions, where the DNA·protein interactions are stabilized by cellular polyamines and the surface is not coated with a high concentration of polycations, while still maintaining enough contact to be imaged. This approach allows us to simplify the sample preparation protocol (no surface pretreatment) and increase the observation time to minutes compared to that in previous HS-AFM reports,^29−32^ as will be discussed below. Lengthening the lifetime of the molecules was critical because the observation of volume fluctuations for several seconds allows its quantification and thus the identification of the disassembly products; furthermore, it facilitates the detection of other dynamic changes due to DNA unwrapping and/or histone dissociation. To characterize nucleosome disassembly products, we trained a neural network (Methods and Figure SI 2B) with >300 images of different molecules in which the DNA and NCP were manually segmented. The resulting algorithm identifies and differentiates the histone core and DNA arms from the background, which allows us to track the NCP volume and the dynamics of each DNA arm at the entry and exit sites by reporting their angular changes (Figure 1B(i) and (ii) and Movie SI 1). The measured volume values are also in agreement with those found in the literature when scanning in liquid.^27,31^ Together, the NCP volume and the asymmetric length of the DNA arms allow us to unequivocally distinguish intact nucleosomes from PANS (Figures 1A and SI 3A and SI 5A), although we acknowledge that this chosen geometry, may primarily represent those nucleosomes situated at the termini of chromatin arrays, potentially featuring free DNA ends. Unfortunately, we were unable to quantitatively assess the length of unwrapping during the protein dissociation events since the DNA length could not be measured accurately frame-by-frame due to its high mobility and occasional partial or total invisibility due to transient desorption from the mica surface (Movies SI 2 and SI 3). The extent of nucleosome unwrapping has also been assessed by measuring DNA opening angles (defined by the vectors connecting the intersections between the DNA arms and the NCP-enclosing ellipsoid to the center of that ellipsoid).^28^ However, we refrained from using this approach because we realized that these angular values result from the convolution of the extent of nucleosomal DNA wrapping plus the orientation adopted by the complex during its adsorption onto the surface (see SI).

Most in vitro experimental approaches have limitations that affect the results, and surface microscopy techniques such as AFM are no exception. As a result, in the Supporting Information, we review the effects of high-speed scanning and the mica surface on nucleosome disassembly. Furthermore, we performed control experiments to emphasize that the observations described here are mostly intrinsic to nucleosome dynamics and are not dictated by our experimental conditions. Namely, we demonstrated that inhibiting nucleosomal DNA unwrapping or large NCP rearrangements prevents nucleosome disassembly, as will be discussed further below. Nonetheless, during extensive scanning of individual molecules, occasional interactions between the scanning tip and DNA arms may induce nucleosome unwrapping, potentially catalyzing their disassembly.

High-speed AFM imaging shows the disassembly of single nucleosomes moving in a 2D space where their NCP appears quasi-spherical due to the tip convolution and the DNA arms move freely in and out of the surface (Figure 1A frames 2, 3, 6, and 7 and Movie SI 2). This dynamic behavior facilitates nucleosomal breathing (temporarily exposing nucleosomal DNA through spontaneous unspooling from either end) or DNA unwrapping, which presumably triggers its disassembly. The height of the NCP undergoes small fluctuations over time until sudden changes in height are observed, which we interpreted as a disassembly event and that are almost invariably irreversible (Figure 1A frames 3–6 and 5). The nucleosome fully disassembles in about 50 s, leaving bare DNA (Figure 1A frame 12). To further confirm this interpretation, we computed the volume of the NCP over time and observed a stepwise volume decrease consistent with the formation of subnucleosomal particles (Figure 1B top (i) and middle panels). We proposed that the stepwise changes in the nucleosome’s volume correspond to the sequential dissociation of one and then the other H2A·H2B heterodimers, forming a hexasome and a tetrasome, respectively. The loss of the two heterodimers is followed by the dissociation of one H3·H4 heterodimer, yielding a disome and finally bare DNA (Figure 1B middle panel). The sequential disassembly that we visualize here at subsecond time resolution corroborates the models proposed by sm-FRET,^16^ time-resolved SAXS,^15^ and atomistic molecular dynamics.^13^

The nucleosomes undergo conformational fluctuations in which the NCP transitions from spherical shapes to oblong, even lobed, morphologies for a variable amount of time before a heterodimer dissociation event occurs (Figures 1A,C and SI 3B and Movies SI 2 and SI 3). The lifetimes of nucleosomes and subnucleosomal particles vary significantly (from a few seconds to minutes) (Figure 1C), even when they are imaged simultaneously in the same frame (Figure SI 3B). As previously noted, this phenomenon could be ascribed to the initial structural diversity, encompassing various degrees of DNA unwrapping and NCP conformational states, induced by the sample’s dilution. Conversely, we were not able to estimate tetrasome lifetimes accurately, tetrasomes survive longer times, and we were frequently forced to end the observation—due to the mobility of the molecule out of the field of view or interactions with neighboring molecules—before the tetramer was fully dissociated. These results agree nicely with the high-precision kinetic FRET studies, which demonstrated that the final transition in nucleosome disassembly—tetrasomes—is slow over a time scale ranging from minutes to hours.^16^ Likewise, determining the average duration required for complete molecular disassembly remained elusive, primarily because most of the molecules retained their tetrasome configuration, and we could not always observe the disassembly of these resilient PANS. The fact that tetrasomes endured extended scanning, in contrast to nucleosomes or hexasomes, suggests that, in general, the molecular organization (e.g., lack of DNA wrapping and high (H3·H4)_2_ stability) exerts a dominant effect on the AFM experimental conditions. The AFM-induced destabilization on the nucleosomes will be discussed below.

To determine which side of the nucleosomal DNA unwraps first, we track the angular fluctuations between the DNA arms and the NCP at the entry (θ_2_) and exit (θ_1_) sites of the nucleosome (Figure 1B top (ii) and lower panels and Movie SI 1). Our analysis shows that when the first H2A·H2B heterodimer is ejected, it causes a large change in the angular orientation of the short DNA arm while the fluctuations of the long DNA arm (θ_2_) remain roughly constant (Figure 1B middle and bottom panels, first shaded rectangle). This change is interpreted as an increase in accessible configurations of the DNA due to the disruption of DNA·protein interactions and the release of a region of the outer nucleosomal DNA wrap. The conversion of nucleosomes to hexasomes during the first H2A·H2B dissociation event is characterized by a decrease in volume of ∼200 nm^3^ and an increase in the length of the short DNA arm. This observation indicates that during nucleosome disassembly the distal heterodimer is the first to dissociate from the rigid nucleosomal DNA side. Moreover, as shown in the bottom panel of Figure 1B, while the angular fluctuations of the long DNA arm (θ_2_) remain roughly constant at the time of the first dimer ejection, the short DNA arm at the exit site (θ_1_) undergoes a change of ∼90°. In contrast, the ejection of the second H2A·H2B dimer occurs on the opposite and flexible side of 601 NPS, inducing now the angular change in θ_2_ of ∼55° (1B bottom panel). Figure 1D highlights the structural changes that the nucleosome and hexasome undergo after each H2A·H2B dissociation event. To facilitate the visualization of the changes observed by AFM, we added a cartoon representation keeping the nucleosome’s left-handed chirality and the (H3·H4)_2_ at the dyad. This representation which matches the AFM data for the transition from nucleosome to hexasome shows the short DNA arm lengthening (green) and its corresponding angular change as well as a slight rotation of the histone core when the distal H2A·H2B heterodimer dissociates (Movie SI 2). In contrast, during the transition from hexasome to tetrasome, the angular change of the long DNA arm (orange) is smaller, and no histone core rotation was observed when the proximal heterodimer was ejected. Once the tetrasome forms, both DNA arms exhibit similar lengths and are relatively parallel to each other (Figures 1A frames 7–9 and SI 5A, first row 25 s; Table SI 1). They continue to fluctuate on the surface without noticeable DNA angular changes during tetrasome and disome disassembly (Figure 1B bottom panel).

As described in the Methods section, the intricate set of concurrent dynamic events captured in each frame obligates us to limit the angular analyses to frames in proximity to dimer ejection events to further validate our observation. Figure SI 4A illustrates a consistent angular change, primarily at or near the exit site (short arm) of the nucleosome core during the transition from nucleosome to hexasome. In contrast, angular values at the entry site (long DNA arm) remain relatively stable. Conversely, during the transition from hexasome to tetrasome, the opposite behavior is observed, with minimal angular changes at the exit site compared to pronounced changes at the entry site, as depicted in Figure SI 4B. It is worth noting that the precise timing and magnitude of these angular changes vary across different nucleosomes, likely due to the diversity in the orientations and conformational state of the nucleosomes on the surface at the instant of the ejection convoluted with variations in the extent of DNA wrapping around the histone core. Furthermore, this closer examination also reveals that NCP volume changes can manifest in two different manners: (1) a sudden drop within 1 s (Figure SI 4A nucleosomes 1, 3, and 5) or (2) a gradual decay over 1 to 2 s (Figure SI 4A nucleosomes 2 and 4). This is probably related to the way that the dimers dissociate from the core.

Overall, our findings suggest a coupling between DNA unwrapping and dimer dissociation and support the asymmetric model of nucleosome unwrapping^15−18^ at physiological ionic strength and temperature and without the application of external force. While the Widom 601 sequence is not a naturally occurring nucleosome positioning sequence, it has been widely utilized in in vitro studies of nucleosomes. These studies have contributed significant insights into nucleosome dynamics, providing valuable information on their structural, biochemical, and mechanistic aspects. Furthermore, the similarity in nucleosome architecture between naturally occurring sequences and the well-studied 601NPS,^43,44^ along with the fact that asymmetry is a common feature in nucleosomes across the entire genome,^4^ lends confidence to the idea that the dynamics observed in this study can be extrapolated to biologically relevant scenarios. This methodology offers promising avenues for investigating the roles of diverse positioning sequences, histone variants, and epigenetic modifications.

**Figure 2:**
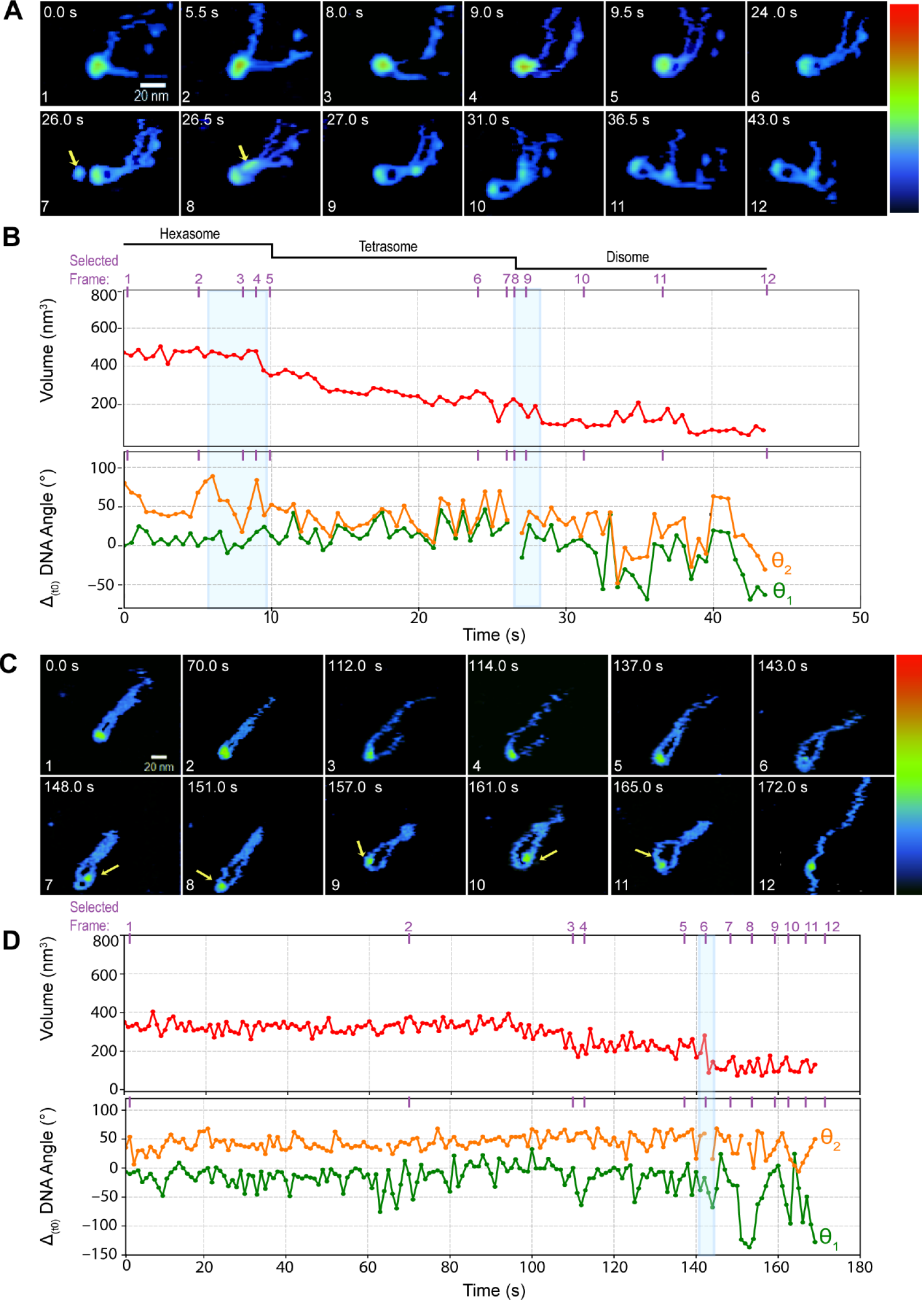
Dynamic disassembly of purified subnucleosome structures. (A) Time excerpts and (B) time course of volume (top) and DNA arms angles (bottom) changes caused by the disassembly of a purified hexasome. Histone reversible ejection is indicated by the yellow arrow. (C) Time lapse and (D) volume (top) and DNA linker angular evolution (bottom) of a de novo tetrasome. Angular fluctuations are relative to the value at time zero (Δ _(t0)_), traces corresponding to θ_2_ were offset upward for clarity. The movement of the (H3·H4) is depicted by yellow arrows in C. Z-color-map from 0 to 5.5 nm. Purple ticks and numbers in the plots show the selected time point displayed in the images. H = hexasome, T = tetrasome, and D = disome. Blue-shaded rectangles highlight the simultaneous DNA angle and NCP volume changes during a dimer eviction.

To confirm PANS assignments (hexasomes and tetrasomes), we characterize their dynamics as purified particles (Figure SI 1A,B). As observed in Figure SI 1A, the purification of hexasomes is highly efficient, the number of entire nucleosomes is negligible, and the small fraction of bare DNA still present in the sample was avoided during the AFM imaging. The morphology and dynamic disassembly of purified hexasomes and those originated from H2A·H2B dimer dissociation during our observations are very similar; their initial volume is ∼200 nm^3^ smaller than that of nucleosomes and their DNA arms are of similar length. This observation suggests that the result of the first dimer’s dissociation is independent of the experimental conditions by which it is attained and confirms that the asymmetric disassembly is initiated at the distal dimer side (Figure 2A, Figure SI 5A second row, and Movie SI 4). Purified hexasomes follow a histone dimer-stepwise disassembly trajectory like that of nucleosomes (Figure 2A frames 5–12 and 2B top panel). Our time lapse also shows that dimer dissociation is dynamic; a dimer that has left the core can rebind to the DNA before gliding away (Figure 2A frames 7–9 and Movie SI 4). We also characterized the dynamic disassembly of de novo tetrasomes assembled with pure H3·H4 tetramers (Figure 2C and Movie SI 5). Compared to purified nucleosomes and hexasomes, de novo tetrasomes are long-lived and exhibit additional dynamics described in more detail in the following section.

The AFM movies allowed us to identify DNA configurations previously inferred by TR-SAXS and FRET,^15^ including the formation of the teardrop DNA open intermediate geometry (Figures 1C Nuc 2 at 65 s and 2A frame 8 and SI 3B 41.5 s). Additionally, we observed at least two different mechanisms for dimer dissociation. In the more common mechanism, the dimer transiently separates from the NCP, which sometimes reversibly transitions from a round to a more lobed shape (Figure 1C Nucs 2, 3, and 5). After a brief period (typically less than 2 s), the heterodimer fully dissociates from the core and diffuses away over the DNA (Figures 1A frames 11–12 and SI 3 and Movies SI 2 and SI 3). Occasionally, the dimer dissociates from the core and temporarily interacts with the surface before reattaching to one DNA arm to slide away along it (Figure 2A frames 7–8 yellow arrows, Movie SI 4). In the second mechanism, the heterodimer is simply ejected from the core and disappears from the field of view (Figure 1A frame 10 yellow arrow). These observations are also consistent with the two modes of volume change described above (Figure SI 4A).

### H3·H4 Structures Diffuse Freely on the Nucleosome Positioning Sequence

As stated above, we observed that tetrasomes, whether produced by nucleosomal disassembly or de novo, are long-lived compared to nucleosomes and hexasomes. Additionally, tetrasomes have often been reported to survive the passage of RNA polymerases (Pol II) through nucleosomes.^45−47^ Since the (H3·H4)_2_ complex is the first to bind to DNA during de novo assembly of nucleosomes both in vitro and in vivo,^48,49^ we aimed to compare the dynamics and lifetime of tetrasomes formed through disassembly with those formed only with H3·H4 dimers. This comparison could allow us to determine if interactions with H2A·H2B heterodimers that have been disrupted affect the dynamics and lifetime of tetrasomes formed through nucleosome disassembly. We were able to observe de novo tetrasomes for ∼2 min, which is about 6 times longer than that of nucleosomes.

While it took this time to dislodge one H3·H4 dimer from the complex (Figure 2D frame 6), the second dimer remained bound and diffused along the curved segment of the DNA (Figure 2C,D frames 7–11 top panel) throughout the entire observation time (∼3 min) (Figure 2D frames 7–11 top panel). We note that DNA arms of de novo-formed tetrasomes are mainly parallel and very close to each other and do not exhibit significant angular variation before H3·H4 dimer dissociation (Figure 2C,D bottom panel), a feature also exhibited by tetrasomes generated through nucleosome disassembly (compare tetrasomes in Figures 1A,C, 2A, and SI 5A with those in Figure 2C).

Surprisingly, we observed that the length of the two DNA arms in tetrasomes produced during nucleosome disassembly was similar (Figures 1A, 2A, SI 3B, and SI 5A). Our intentionally asymmetrically designed nucleosomes had an expected DNA arm ratio (*R*_DNA_) of ∼2:1 (entry:exit sites; Figure SI 5B top panel). This ratio decreases to 1.5:1 after the first heterodimer dissociation (Figure SI 5B middle panel). However, if ∼35 bp of DNA are released on each side of the histone core after each dimer dissociation and the remaining tetramer does not move from its original position (Figure SI 5B bottom panel), then the ratio should be partially restored. Under this assumption, we expect *R*_DNA_ values of ∼1.9:1 for nucleosomes and ∼1.7:1 for tetrasomes. These values are partially affected by the precision of our measurement methodology (Figure SI 5B). To estimate the variation of *R*_DNA_ of tetrasomes resulting from nucleosome disassembly (Figures 1A and 2A), we measured the length of the DNA arms on a few frames where they were clearly and fully visible (Figure SI 5A and Table SI 1). We found that the sum of the DNA arms’ length is ∼116 nm for nucleosomes and ∼133 nm for tetrasomes and that *R*_DNA_ varies from 1.6:1 in nucleosomes to 1:1 and 1.3:1 in tetrasomes (Table SI 1). This result confirms our observation that (H3·H4)_2_ slides along the 601 NPS sequence during nucleosome disassembly.

**Figure 3:**
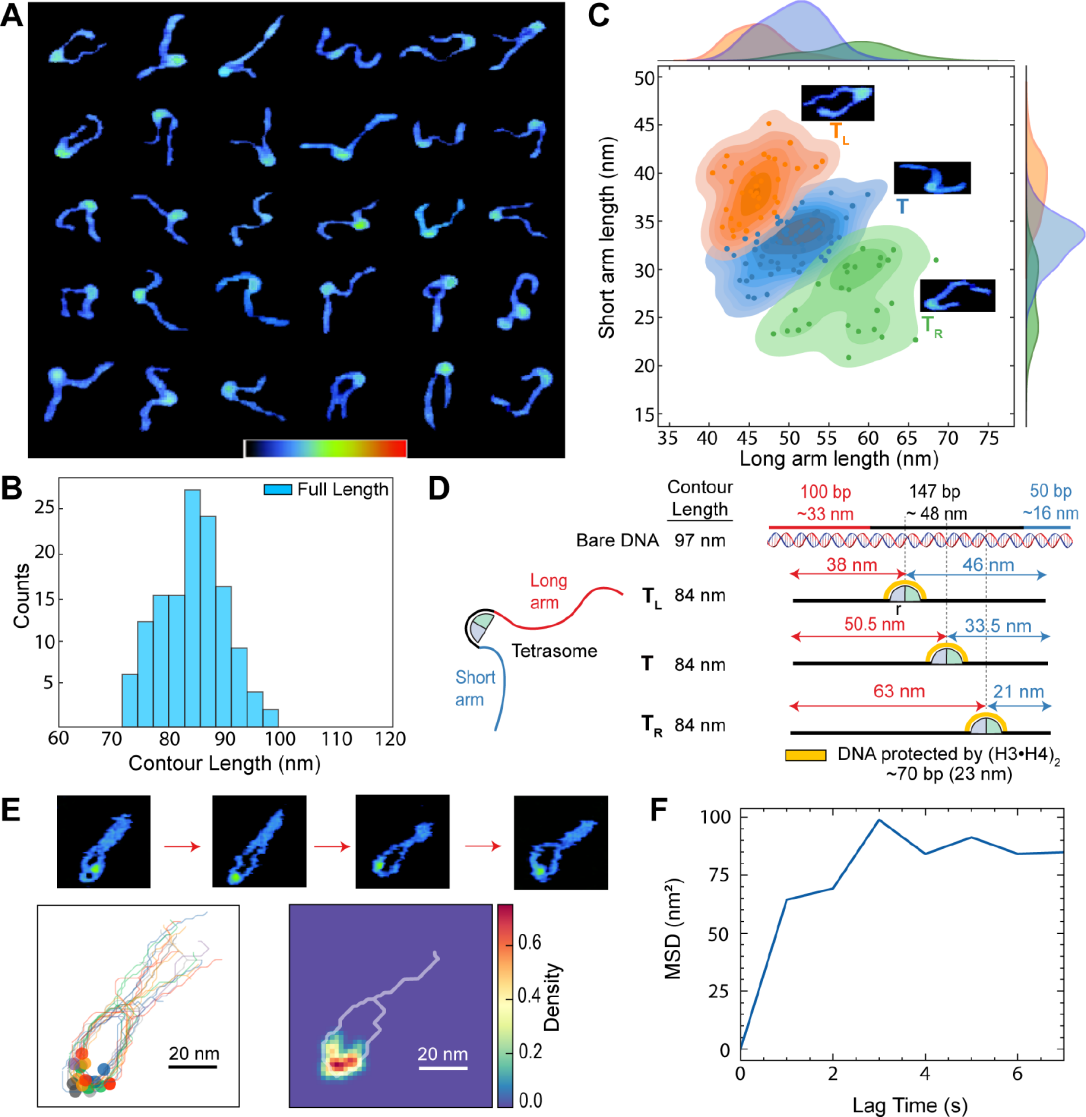
Positioning of histone tetramer in de novo tetrasomes (**A**) Six representative tetrasome molecules assembled on a shorter DNA template (columns) from five different AFM micrographs and two different depositions (rows). Z-color-map from 0 to 5.5 nm. (B) Distribution of measured DNA full length of 130 tetrasomes extracted from AFM micrographs. The mean DNA contour length is (84.0 ± 5.8) nm, error is the SD (C) Distribution of the long DNA arm (top) and the short DNA arm lengths (right) and their correlation. Colored dots and contour kernels represent three tetrasome states identified by the *k*-means clustering algorithm. Insets: AFM images illustrating different positions of the (H3·H4)_2_ tetramer. (D) Representation of the short DNA template (100W50) used to assemble de novo tetrasomes with its expected dimensions in nanometers (top). Hypothetical DNA dimensions of three tetrasome configurations determined by the location of the (H3·H4)_2_ and adjusted to our measurement methodology (see Methods; DNA length plus the histone core radius *r* ≈ 5 nm). In the T configuration, (H3·H4)_2_ is positioned around the 601 NPS central region, whereas in the T_L_ and T_R_ configurations (H3·H4)_2_ is located at the left or right side of the 601 NPS center, respectively. (E) AFM images displaying the histone displacement within the de novo tetrasome featured in Figure 2C (top). The real-time positions of the histones were tracked for 40 s, with their locations represented by colored circles. To enhance clarity, the DNA traces were aligned (bottom-left), and a heat map (bottom-right) reveals the preferred histone occupancy within the 601 NPS. (F) The mean square displacement of the tetrameric histones, as depicted in (E).

In our real-time observation of de novo tetrasome dynamics, the length of the DNA arms visibly fluctuates due to the reversible displacement of the histones over the DNA (compare the position of the histone core in Figure 2C frames 1–5 with frames 7–10 yellow arrows). We also detected a confined diffusion of the histones in the 601 NPS region when the two DNA arms were separated as shown in Figures 2C,D and 3E,F. A superposition of tetrasome DNA traces and their respective core position shows that the histones can occupy various positions around the NPS, with a preference for the center of the curvature, as depicted in the heat map (Figure 3E bottom panel). The analysis of mean square displacement for histones in frames with detectable movement indicates that their diffusion is confined and that it levels off at approximately 80–90 nm^2^, suggesting a displacement of nearly 10 nm in less than 10 s (Figure 3F). Detecting this dynamic proved to be challenging since, in most observations, the DNA arms of the tetrasomes remained in interaction with one another in a closed configuration (Movie SI 6). This configuration adopted by the DNA within the tetrasomes may render them resistant to disassembly. Nevertheless, the diffusion of tetrasomes on DNA has also been reported by Katan et al.^29^ To determine if the movement of the tetramer is a product of the nucleosome disassembly or is an intrinsic property of the tetrasome, we image larger areas crowded with de novo tetrasomes. As shown in Figure SI 5C, a visual inspection of the DNA arms’ length of different molecules depicts high variability, confirming that it is a property of (H3·H4)_2_ to diffuse along the 601 sequence.

To quantitatively determine the most probable binding site of (H3·H4)_2_ in de novo tetrasomes, we assembled tetrasomes on the 601 NPS with shorter but still asymmetrical DNA arms (100 bp flanking the flexible side and 50 bp flanking the rigid side, 100W50) and imaged them in air to accurately measure the length of their DNA arms (Figure 3A). This construct with short DNA arms facilitated the data analysis. Like our longer construct, the length of the DNA arms is highly variable and the distribution of the sum of both DNA arms agrees well with the expected DNA length of this molecule (Figure 3B). We then determine the preferred location of the tetramer by plotting the length of the short arm against the length of the long DNA arm.

We observe a broad, weakly anticorrelated, and somewhat fragmented scatter pattern (Figure 3C), which indicates the presence of several populations of tetrasomes due to the variable position of (H3·H4)_2_. We used the *k*-means clustering algorithm to determine the probability of finding subpopulations of tetrasomes. Indeed, as shown in Figure 3C, there are at least three probable locations of the tetramer along the NPS. To explain these three populations, we calculated the expected DNA arm lengths when the tetramer is located at the center of the 601 NPS or shifted to the left or right side (Figure 3D T, TL, and TR, respectively). These calculations agree with the experimental populations. The larger fraction of molecules (∼55%) corresponds to the central location (T), followed by T_L_ (∼26%) and T_R_ (∼20%). This result indicates that although (H3·H4)_2_ can diffuse along the NPS within a range of 12 to 15 nm (∼35–45 bp, equivalent to the DNA protected by a single histone dimer) it tends to remain around the central region of 601 NPS and that the probabilities of diffusing toward the flexible or rigid side of the 601 NPS sequence are very similar.

It is thought that the directional displacement of nucleosomes and hexasomes along the DNA requires ATP-dependent chromatin remodelers, although modest levels of reversible nucleosomal sliding in the absence of remodelers have been reported.^32,50^ In this study, we observe that tetrasomes can easily diffuse over the DNA without external energy sources. The mobility of the tetramer could be due to the lack of full DNA wrapping around the subnucleosomal particles, which would otherwise constrain the motion in nucleosomes and hexasomes. Moreover, the mobility of (H3·H4)_2_ around the central region of 601 NPS could be a mechanism to tune subsequent H2A·H2B binding events and proper DNA wrapping and thus favor a specific structure and stability (i.e., canonical-stable nucleosomes, hexasomes, noncanonical nucleosomes, etc.). To the best of our knowledge, there is no experimental evidence to support that this is the case. However, molecular dynamics simulations have predicted that during nucleosome assembly the probability of attaining the crystallographic canonical structure of a nucleosome depends on the preferential initial binding site—left or right—of the H2A·H2B heterodimers relative to the position of (H3·H4)_2_ on the NPS.^51^ This same study also reveals that nucleosome assembly displays a rugged kinetic landscape populated with canonical and noncanonical nucleosome intermediates.^51^

### Dynamic Disassembly of Chromatosomes in Real Time

The linker histone H1 acts as a condenser of chromatin in repressive heterochromatic genomic regions by binding tightly to nucleosomes at their entry–exit sites. H1 binding stabilizes NCP interactions and promotes internucleosomal connections.^8,52^ Structural studies have revealed that H1 binding causes the formation of an apposed linker DNA stem motif (i.e., ∼10 bp of each flanking DNA linker are brought into close proximity, forming a stem-like structure), which restricts DNA unwrapping so that the nucleosome adopts a more compact and rigid structure known as a chromatosome.^33,39^ To determine how these structural and functional differences between nucleosomes and chromatosomes affect their disassembly dynamics, we assembled chromatosomes using the human linker histone H1.0 and monitored their dynamics by HS-AFM. The binding of H1.0 to nucleosomes results in a noticeable shift in the migrating band of the nucleosome, along with the bands associated with hexasomes and free DNA (Figure SI 1C, left panel). In contrast to nucleosomes, the purification of this sample primarily yields chromatosomes (80%), providing further evidence of the stabilizing role of the linker histone (Figure SI 1C, right panel).

Whereas the nucleosomal NCP appears to be mainly spherical (Figure SI 6A), the chromatasomal one exhibits a teardrop morphology, which we attribute to the linker DNA stem (Figure SI 6B white circles). We determined that the volume distributions of the histone core of nucleosomes and chromatosomes are broad and similar (Figure SI 6A,B). The volume of surface maps of nucleosomes and chromatosomes are not too different even when comparing structures solved at high resolution^53,54^ (Figure SI 6C, top row). The volume expected from AFM images of these structures simulated with the resolution comparable to those obtained in this study is within the range of our measurements (Figure SI 6C bottom row). However, the chromatosome’s volume distribution fits well to a normal distribution, while that of the nucleosome fits better to a trimodal distribution (Akaike information criterium, AIC) (Figure SI 6A,B bottom panels). We inferred that the presence of nucleosomes and hexasomes contributed to the observed trimodal distribution, with a predominant species with a broader conformational variability in chromatosomes. This result aligns well with the findings from the electrophoretic analysis (SI, Figure SI 1A,C). For instance, the largest nucleosome population displays volumes corresponding to octameric cores organized similarly to the known canonical structures (365.4 ± 49 nm^3^) whereas the smaller volume fraction could represent hexameric cores and the fraction with larger volumes could represent nucleosomes with different degrees of DNA unwrapping and/or structural conformations.^28,55^ Variations in volume can arise from different orientations adopted by the complexes during the adsorption onto the surface. Thus, it is possible that H1 not only stabilizes the NCP and hinders DNA unwrapping but also, in doing so, increases the dynamics of the histone core, potentially promoting a greater number of conformational states. A similar mechanism has been proposed for the heterochromatin protein HP1.^56^

**Figure 4:**
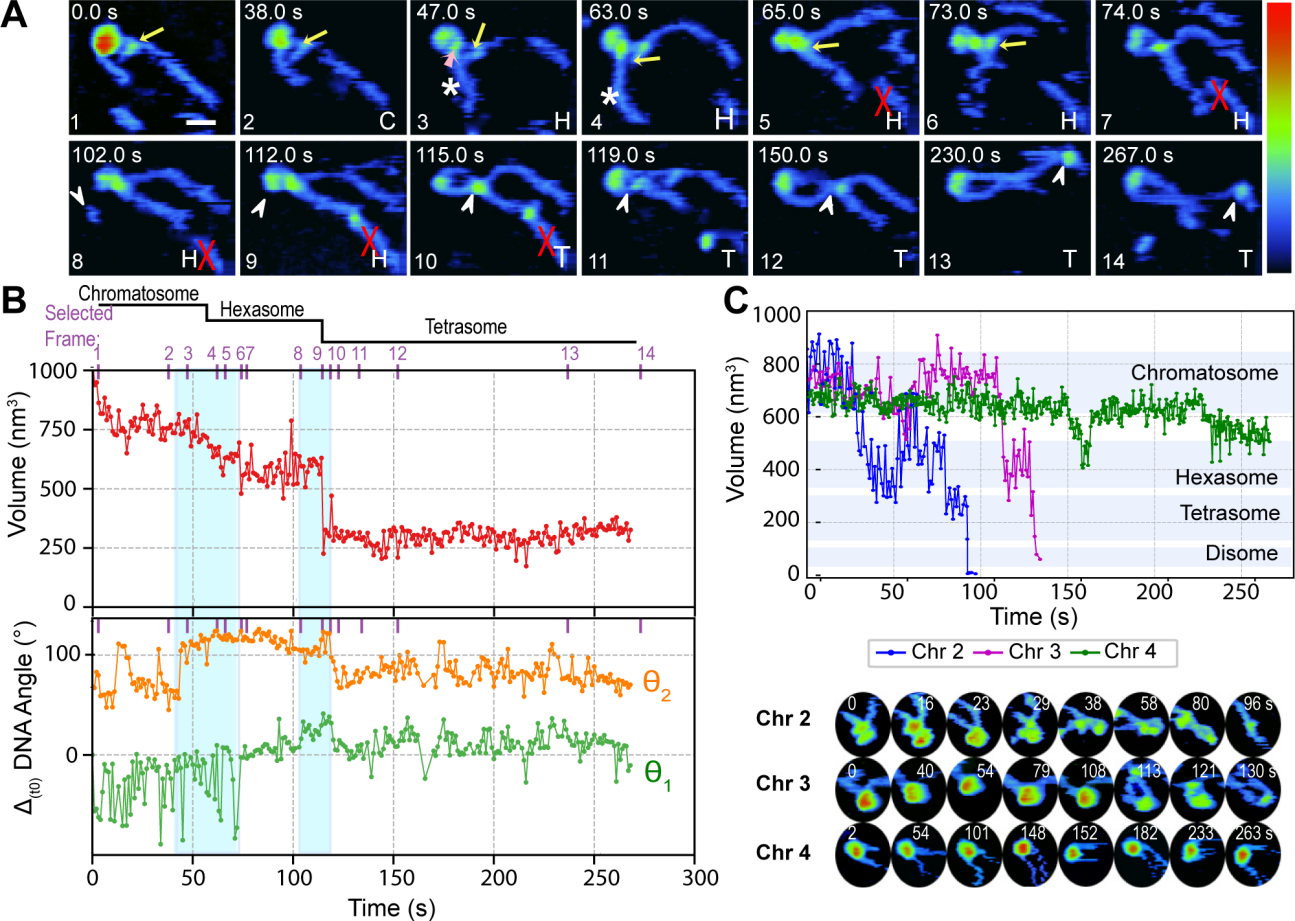
Chromatosome dynamic disassembly. (A) Time lapse of a chromatosome disassembly. Yellow arrows point out the dynamics of the linker histone H1, the pink arrow indicates a gap in the core due to the eviction of a heterodimer, white asterisks specified the lengthening of the short DNA arm, and white arrowheads show a reversible ejection event as well as the gliding away of a core histone dimer. The red crosses show a transient intruder DNA from a neighboring molecule. Z color map from 0 to 6 nm. C = chromatosome, H = hexasome, and T = tetrasome. (B) Time traces of the changes in volume (top) and DNA arm angles (bottom) of the molecule in A. Angular fluctuations are relative to the value at time zero (Δ _(t0)_), the trace corresponding to θ_2_ was offset upward for clarity. Purple ticks and numbers in the plots show the time point displayed in the images. (C) Examples of three additional chromatosome volume trajectories as a function of time. Compare observation times of chromatosomes with those of nucleosomes in Figure 1. Shadowed regions indicate the range of volume observed for different disassembled states.

We found that it takes ∼50 s for the chromatosome to disassemble, which is roughly twice as long as for nucleosomes (Figure 4A and Movie SI 7). At the beginning of the process, the NCP of the chromatosome appears spherical but also exhibits a distinct density at its entry–exit site, which we attributed to H1 (Figure 4A yellow arrow and Figure SI 6D).^39^ The apparent gap observed between H1 and the short DNA arm at time zero is partially due to the orientation adopted by the molecule during its adsorption on the surface (Figure SI 6D left panel). It is also probable that the interaction of H1 with this DNA arm is mediated by its long and intrinsically disordered C terminus,^39^ which could be partially extended and therefore difficult to visualize. The histone core shows conformational fluctuations for about 45 s as detected by the evolution of both the NCP’s height (Figure 4A and Movie SI 7) and volume (Figure 4B top panel). During this period, H1 remains bound to both DNA arms, which can only modestly separate, in contrast to that observed for nucleosomes (Figure 1A and SI 3). Notice that within a 47 s interval, we observed the dissociation of one heterodimer from the histone core while H1 remained bound to the DNA linkers (Figure 4A frame 3 and Figure 4B top panel, Movie SI 7). This caused the histone core to adopt a lobe-shaped conformation and its volume to decrease, which was accompanied by a visible increase in the length of the short DNA arm (Figure 4A frame 3 white asterisk and 4B bottom panel). At 63 s, there is a detectable angular change and an additional increment in the length of the shorter DNA arm (Figure 4A,B frame 4 white asterisk). Both DNA arms now appear to be of similar length, indicating that DNA was able to unwrap from the core in the presence of H1. This observation is consistent with the asymmetrical dissociation of an H2A-H2B heterodimer from the rigid side of the 601 NPS, even though the DNA unwrapping and angular changes are different compared to that of nucleosomes, probably due to the presence of the still-bound linker histone (compare Figures 1B and 4B bottom panels). The linker histone can dock reversibly into the core (Figure 4A,B frames 4–6 yellow arrow), and a sudden angular fluctuation (>45°) of the former short DNA arm triggers its ejection within 1 s (Figure 4A frame 7), resulting in a canonical hexasome with a volume in the expected range for this PANS (Figure 4B bottom panel). The hexasome continues to rearrange until the second heterodimer is transiently ejected from the histone core (Figure 4A,B bottom panel frame 8 white arrowheads from Movie SI 7). At 112 s, the heterodimer binds again to the core (frame 9) for ∼10 s before sliding away along a DNA arm (Figure 4A,B top panel frames 10–14 white arrowheads). The resulting PANS remains as a tetrasome with a small volume, nearly parallel DNA arms, and reduced DNA angular changes for the rest of the observation time (∼4.6 min).

Like nucleosomes, chromatosomes also dissociate in a stepwise and asymmetric fashion with a highly variable and stochastic duration (Figure 4C). Nonetheless, the interaction of both DNA arms with H1 greatly reduces their degree of freedom, causing DNA unwrapping to be constrained and effectively eliminating large DNA angular fluctuations. This restriction not only delays disassembly but also results in an alternative disassembly pathway. In other words, it is thought that the dimers dissociate from nucleosomes as a consequence of core rearrangements induced by DNA unwrapping.^55^ When unwrapping is inhibited by H1, thermal and electrostatics fluctuations in the core can still induce a dimer eviction event from the NCP. The two-step lengthening of the short DNA arm, followed by the docking of H1 into the histone core (Figure 4A,B frames 4–6 and yellow arrows) reflects a modified disassembly pathway compared to that of nucleosomes. Thus, H1 modifies the nucleosomal disassembly landscape. However, if, stochastically, H1 dissociation is the first step in the process, then the disassembly of chromatosomes and nucleosomes becomes indistinguishable (Figure 4C, chr 2 and 3).

The disruption of interactions between DNA and histones seems to play a major role in nucleosomal disassembly. Namely, when the DNA arms are steadily interacting with the histone core (e.g., Figure 4A frames 1–2, the DNA arms are parallel to each other with minor angular fluctuations as observed in chromatosomes), the molecules are stable and last longer, presumably because the DNA·protein interactions are only slightly perturbed. Conversely, if as a result of DNA fluctuations these interactions are disrupted, then their disruption may elicit the eviction of histones. Another peculiar feature of chromatosome disassembly dynamics, which are much less common in the disassembly of nucleosomes, is their ability to reverse the ejection of a heterodimer. Namely, dislodged core histones that remain near the NCP (either interacting with the linker DNAs or in the process of diffusing away) have the potential to bind back into the core, which is reflected by the large and reversible volume fluctuations observed in these complexes (Figure 4C). The movie frames at the bottom of Figure 4C (e.g., Chr 2) show the dissociation of a histone dimer and the concomitant formation of a teardrop structure (38 s) that slides back to the core (58 s) on the same DNA fragment, demonstrating that the large variations in volume are indeed due to a rebinding event and not to an intruder molecule (as in Figure 4A) or noise. We speculate that this effect is due to the geometry and restricted motion of DNA arms in chromatosomes, which keeps dissociated dimers in proximity and thus favors reassembly. The disassembly of chromatosomes, much like nucleosomes, experiences a substantial deceleration after transitioning to tetrasomes. Consequently, we faced challenges in accurately quantifying the time required for complete disassembly.

Our volume analysis provides a key advantage by granting real-time insights into the conformational changes within the histone cores of the various molecular species under investigation. We computed volume values between discernible dimer ejection events and plotted their distributions (Figure SI 7A,B). As anticipated, the volume distributions for chromatosomes, nucleosomes, hexasomes, and tetrasomes consistently shift toward lower values (with mean values of 673.7 ± 155.8 nm^3^, 594.8 ± 116.3 nm^3^, 470.2 ± 113.6 nm^3^, and 351.7 ± 74.5 nm^3^, respectively), as depicted in Figure SI 7B. However, the full width at half-maximum of these distributions varies significantly across the species, suggesting a diversity of histone core conformations (Table SI 2). For instance, chromatosomes appear to explore a wider range of conformational states in comparison to nucleosomes, while hexasomes exhibit a variety of morphologies, with a distinct lower volume population possibly associated with more open conformations such as the often-observed teardrop structures. These findings support our earlier qualitative observations where we described reversible transitions in the histone core, shifting from spherical to lobed configurations before disassembly. The broad volume distributions derived from single chromatosomes’ dynamics align nicely with the similarly broad volume distributions obtained when imaging hundreds of static molecules (Figure SI 6B). These dynamic oscillations probably, in part, reflect the intrinsic plasticity of nucleosomes.^6−8,16,53,55−58^ Thus, it is conceivable that subtle rearrangements in the histone core induce changes in protein·protein as well as DNA·protein interactions which, in turn, modulate DNA unwrapping and trigger molecular disassembly.

Next, we calculated the time elapsed before the occurrence of the first histone dimer ejection event and plotted the cumulative distribution function (CDF). This time interval marks the initiation of the disassembly process for chromatosomes, nucleosomes, and hexasomes. We excluded tetrasomes from this analysis since we could seldom observe their disassembly. Figure SI 7C illustrates that both nucleosomes and hexasomes exhibit a similar probability of ejecting a histone dimer within approximately 25 s, with nucleosomes having a visibly higher likelihood of enduring beyond that time. In stark contrast, chromatosomes require twice the time, taking ∼50 s or even up to 2 min for the first dimer ejection event to occur. These results nicely support the concept that linker histones enhance the stability of nucleosomes by reducing DNA flexibility and decreasing the likelihood of NCP unwrapping.^39,52^

Taken together, our findings suggest that the primary mechanism driving disassembly involves the unwrapping of nucleosomal DNA, which subsequently triggers significant core structure rearrangements, facilitating histone dissociation in good agreement with Bilokapic et al.^55^ To investigate whether DNA unwrapping is a prerequisite for histone dissociation, we hindered DNA unwrapping by cross-linking nucleosomes with the reversible primary amine cross-linker, formaldehyde. We observed that while molecular disassembly is impeded in cross-linked molecules, as indicated by their nearly constant volume, the fluctuations in DNA arms can persist (Figure SI 8A,B and Movie SI 8). Notably, despite considerable motion in nucleosome DNA arms, no unwrapping is observed, and the approximately 1:2 ratio in the length of DNA arms remains evident during the observation (Figure SI 8A).

Furthermore, we conducted experiments to explore the impact of increased forces between the AFM tip and nucleosomes on histone ejection. We imaged nucleosomes with stabilized NCPs (i.e., histone·histone cross-linking with dimethyl suberimidate (DMS))^59,60^ for several minutes, during which the tip–sample force was adjusted by varying the amplitude set point. Figure SI 9 shows that the purity of DMS cross-linking nucleosomes was enhanced compared to that of their un-cross-linked counterparts (95% and 71%, respectively). Notably, DMS cross-linking restricts a histone’s mobility without entirely abolishing it, allowing for some degree of nucleosome remodeling by chromatin remodelers, as observed in previous studies.^61^ Indeed, our AFM micrographs further revealed that DMS cross-linked nucleosomes exhibited morphological transitions, including reversible transitions from spherical to oblong and lobed shapes (Figure SI 10A and Movie SI 9), along with more pronounced volume fluctuations when compared to formaldehyde cross-linked nucleosomes (compare Figures SI 10B and SI 8B; RMS = 1099.2 vs 973.7).

Remarkably, nucleosomes with restricted mobility of their histone cores remained intact for several minutes of continuous scanning. Even when force applied by the tip increased, temporarily disrupting the NCP morphology, it rapidly recovered within 1 s, with no histone dissociation events observed (e.g., Figure SI 10A, yellow rectangles in frames 5–6 and 9–10 and purple lines on the volume trace). These nucleosomes also survived a tip disengage–reengage cycle as indicated by the black arrow in the volume trajectory in Figure SI 10B. These results further underscore that the stability of the nucleosome is predominantly influenced by histone·histone and histone·DNA interaction disruptions rather than by interactions with the AFM tip during scanning.

The results obtained in this study present similarities and differences with previous HS-AFM reports.^29,31,32,35,36^ First, the nucleosome disassembly pathway described here consists of a dynamic and yet sequentially controlled series of histone-dimer ejections rather than a one- or two-step process.^29,31,32^ This process may have been missed in previous studies because it was assumed that all complexes remained intact during the sample preparation and/or deposition. We suspect that the material at the start of imaging could have been a mixture of nucleosomes and different PANS. Second, the full stepwise disassembly of a nucleosome occurs in a lapse of minutes, in agreement with sm-FRET results,^15,16^ and not just in seconds as reported by other HS-AFM experiments,^29−32^ even though the scanning rates employed in those studies (1–5 fps) were comparable to our scanning rates (1–2 fps). This difference is likely due to the interactions of DNA with the surface, since in previous studies DNA was attached to a positively charged surface, which favored unwrapped states and hampered rewrapping, ultimately accelerating nucleosome disassembly. In this study, samples were incubated in a buffer with positive multivalent amines to stabilize DNA·protein interactions.^41^ The surface was not precharged, but some residual charges from unbound amines in the buffer cannot be ruled out. This approach allows the DNA arms to be intermittently visible because they can move in and out of the surface, enabling nucleosomal rewrapping and stabilizing the nucleosome against its disassembly. In fact, Melters et al. recently reported that nucleosome mobility decreases significantly when deposited on amino silane-modified mica.^35^ Furthermore, Feng et al. demonstrated that nucleosomes deposited on unmodified mica do not disassemble after extensive high-speed scanning (>5 min) when DNA unwrapping is prevented by binding the nucleosomes’ DNA arms to a DNA origami frame.^34^ Conversely, local interactions of the nucleosomal DNA with positively charged surfaces might weaken the intrinsic flexibility and bendability of the 601 NPS, resulting in an unintended and uncontrolled alteration of the side where the unwrapping will be initiated as reported.^28^ Our findings, combined with those published by others, suggest that nucleosome disassembly results from the synergistic influence of the specific molecule’s composition, structure, and elasticity and the AFM environment. A crucial factor may be the heterogeneity in the nucleosome’s structure, induced by the substantial dilution necessary for HS-AFM, resulting in varying degrees of wrapping and subsequent histone core rearrangements, which, to a certain extent, dictate its stability on the mica. Lastly, we did not observe the DNA loop extrusion that has been reported when nucleosomes were adsorbed on positively charged substrates.^29,32^ Instead, we observed teardrop structures previously proposed by TR-SAXS.^15^

In summary, the AFM data presented here allow us to directly visualize the dynamics of DNA and histones during nucleosome and chromatosome disassembly, providing a simultaneous observation of DNA unwrapping and histone dissociation. Our data strongly support the following conclusions: (1) Asymmetric nucleosome unwrapping and its consequent disassembly can occur under physiological ionic strength and temperature without the need for directional force. We confirmed that DNA unwrapping indeed starts from the rigid side of the 601 NPS, conferring polarity to spontaneous nucleosome disassembly, even with linker histone H1 present. (2) Spontaneous nucleosome disassembly is a dynamic multistep process that proceeds by the sequential ejection of histone heterodimers from the core. Dissociated heterodimers can keep interacting with the DNA and have the potential of binding back to the core. (3) While the (H3·H4)_2_ tetramer predominantly resides within the dyad region of the 601 NPS, it can potentially diffuse a distance equivalent to that occupied by one histone heterodimer on either side of the dyad, with a similar probability. Our study provides new insight into the disassembly of the nucleosomes at the ends of chromatin arrays and the stability of its intermediaries (i.e., PANS), helping to elucidate the mechanisms by which DNA regions become accessible to enzymes such as RNA polymerases, transcription factors, chromatin remodelers, etc.^13,25^ The experimental and analytical strategy presented shows that real-time HS-AFM is a robust and powerful tool for studying single nucleosomes and chromatin dynamics.

## Methods

### Histone Expression and Purification

Recombinant human histones H2A, H2B, H3.3, and H4 were expressed in *E. coli* BL21(DE3) and purified from inclusion bodies as previously described.^17,62^ Histones were centrifuged to remove aggregates, concentrated by centrifugation (∼10 mg/mL), lyophilized, and stored at −80 °C.

### DNA Templates

The DNA template consists of a 601 NPS flanked on the left by 200 bp DNA and on the right by 100 bp DNA (200W100; 447 bp). 200W100 was amplified by PCR from the PGEM 601 vector, and it was cloned back into the PGEM 601 vector using primers containing the restriction recognition site for *BsaI* (NEB). Plasmid containing 200W100 was grown in *dam*^*–*^*/dcm*^*–*^*E. coli* (NEB), purified by maxiprep, and excised by restriction with *BsaI*. The 200W100 template was purified from the vector backbone by 5% preparative acrylamide electrophoresis using a model 491 prep cell (Bio-Rad). An equivalent but shorter DNA template (100W50; 297 bp) was also produced to assemble (H3·H4)_2_ tetrasomes.

### Octamer Reconstitution

The synthesis of the human histone octamer was performed as previously described.^17,62^ Individual histones were dissolved in unfolding buffer (20 mM Tris-HCl buffer pH 7.5, 7 M guanidinium hydrochloride, and 10 mM DTT), and H2A, H2B, H3.3, and H4 histones were combined in a stoichiometric ratio of 1.2:1.2:1:1, respectively, to a final concentration of 1 mg/mL. Unfolded histones were dialyzed for 12 h, using a 3.5 kDa dialysis membrane, against 500 mL of refolding buffer (10 mM Tris-HCl buffer pH 7.5, 2 M NaCl, 1 mM EDTA, and 5 mM BME). This dialysis was repeated four times. Refolded histone octamer was concentrated to ∼0.5 mL using a 10 kDa Ultra-15 membrane filter (Milipore) and fractionated using the Superdex 200 Increase 10/300 GL (Cytiva) gel filtration column equilibrated with refolding buffer. Fractions were analyzed by 15% SDS-PAGE and AcquaStain protein staining (Bulldog Bio), and then fractions containing the octamer were pooled and concentrated to ∼10 mg/mL by centrifugation (30 kDa Ultra-15 (Milipore)). Aliquots were flash-frozen and stored at −80 °C for subsequent use. The synthesis of the (H3·H4)_2_ tetramer followed the same procedure utilized for octamer reconstitution, but H3 and H4 were combined in a ratio of 1:1.

### Synthesis and Purification of Nucleosomes, Hexasomes, Tetrasomes, and Chromatosomes

Nucleosomes, hexasomes, and tetrasomes were synthesized as described by Díaz-Celis et al.^17^ To assemble nucleosomes and hexasomes, histone octamers and a 200W100 DNA template (or 100W50) were combined in a ratio of 1:1, respectively, in 500 μL of high-salt buffer (10 mM Tris-HCl pH 8.0, 2 M NaCl, 1 mM EDTA, 0.5 mM DTT, and 1 mM PMSF) to a final concentration of 100 ng/μL of DNA. Assembly solutions were dialyzed against 500 mL of high-salt buffer for 1 h at 4 °C, followed by linear gradient dialysis against 2 L of low-salt buffer (10 mM Tris-HCl pH 8.0, 1 mM EDTA, 0.5 mM DTT, and 1 mM PMSF) using a peristaltic pump with a 0.8 mL/min flow rate and continuous stirring. A final dialysis of 3 h in 500 mL of low salt buffer (no PMSF) was used to reduce the residual NaCl concentration, and the nucleosome reconstitution was checked with 4% acrylamide native electrophoresis using 0.2X TBE (Tris-borate-EDTA) as a running buffer. Human tetrasome was assembled by combining 200W100 or 100W50 DNA with (H3·H4)_2_ tetramers in a ratio of 1:1.4. The procedure followed for this assembly was the same as that used for nucleosome assembly. Nucleosomes were separated from hexasomes and bare DNA by 4% preparative acrylamide (59:1 acrylamide:bis(acrylamide)) electrophoresis using a model 491 prep cell (Bio-Rad). The prep cell was run at 6 W, and after 1 h, 0.9 mL fractions were collected in 10 mM Tris-HCl pH 8.0, 1 mM EDTA, and 1 mM DTT at a flow rate 0.3 mL/min. Purifications were checked by 4% acrylamide native electrophoresis, and sets of fractions containing nucleosomes or hexasomes were separately concentrated by centrifugation using 100 K Amicon Ultra filters (Millipore). After concentration, samples were dialyzed against HE buffer (20 mM HEPES pH 7.5, 1 mM EDTA, and 1 mM DTT) and stored at 4 °C. For tetrasome purification, reconstituted tetrasome was loaded into 4.8 mL of a 5–20% lineal sucrose gradient (20 mM HEPES pH 7.5, 1 mM EDTA, and 1 mM DTT) and centrifuged for 16 h at 38,000 rpm at 4 °C using a Beckman Optima MAX-XP ultracentrifuge with an MLS-50 rotor (Beckman Coulter). The gradient was fractionated into 100 μL fractions using the Brandel gradient fractionator, and the resulting purification was checked by 4% acrylamide native electrophoresis. Fractions containing tetrasomes and low free DNA were combined, concentrated, and dialyzed against 20 mM HEPES pH 7.5, 1 mM EDTA, and 1 mM DTT.

Human chromatosomes were assembled by a two-step dialysis procedure. First, histone octamers and a 200W100 DNA template were combined in a ratio of 1:1, respectively, in 500 μL of high-salt buffer (10 mM Tris-HCl pH 8.0, 2 M NaCl, 1 mM EDTA, 0.5 mM DTT, and 1 mM PMSF) to a final concentration of 100 ng/μL of DNA. Assembly solutions were dialyzed against 500 mL of high-salt buffer for 1 h at 4 °C, followed by lineal gradient dialysis against 2 L of 10 mM Tris-HCl pH 8.0, 0.6 M NaCl, 1 mM EDTA, 0.5 mM DTT, and 1 mM PMSF using a peristaltic pump with a 0.8 mL/min flow rate and continuous stirring. Second, for human H1.0 incorporation, an increasing molar excess of histone H1 relative to 200W100 nucleosome was added and further dialyzed against 10 mM Tris-HCl pH 8.0, 0.6 M NaCl, 1 mM EDTA, 0.5 mM DTT, and 1 mM PMSF for 3 h, followed by a final dialysis step in HE buffer (10 mM HEPES pH 8.0 and 0.1 mM EDTA) for 4 h. Chromatosome reconstitution was checked by 4% acrylamide native electrophoresis using 0.2X TBE (Tris-borate-EDTA) as a running buffer. Chromatosomes assembled using a ratio of 1:4 DNA:chromatosome were purified by ultracentrifugation for 16 h at 38,000 rpm at 4 °C using a 5–30% lineal sucrose gradient (20 mM HEPES pH 7.5, 1 mM EDTA, and 1 mM DTT). The gradient was fractionated into 100 μL fractions using the Brandel gradient fractionator, and the resulting purification was checked by 4% acrylamide native electrophoresis. Fractions containing chromatosomes and low subspecies and free DNA were combined, concentrated, and dialyzed against 20 mM HEPES pH 7.5, 1 mM EDTA, and 1 mM DTT.

### Cross-Linking of Nucleosomes

Nucleosomes samples were cross-linked with 1% formaldehyde for 1 h at room temperature. Cross-linking reactions were quenched by adding 20 mM glycine for 10 min at room temperature, dialyzed against 20 mM HEPES pH 8.0, 1 mM EDTA, and 1 mM DTT, and centrifugated at 20,000*g* for 10 min to remove aggregates. Formaldehyde is a reversible primary amine cross-linker known to cross-link DNA·DNA as well as DNA·proteins.

We used dimethyl suberimidate (DMS) to selectively cross-link lysines of the histone core of *X*. *laevis* nucleosomes as previously described.^63^ Assembled nucleosomes in 10 mM Tris-HCl pH 8.0, 1 mM EDTA, and 0.5 mM DTT were dialyzed against 500 mL of 100 mM sodium borate pH 10, with two extra buffer changes. After dialysis, the nucleosome volume was determined, and DMS stock (20 mg/mL in 100 mM sodium borate pH 10) was added to the nucleosome solution at a final concentration of 2 mg/mL. After incubating for 1 h, the reaction was quenched by adding 100 mM Tris pH 8.0. The cross-linked nucleosomes were then dialyzed against 10 mM Tris-HCl pH 8.0, 1 mM EDTA, and 1 mM DTT. Cross-linked nucleosomes were purified by 4% preparative acrylamide (59:1 acrylamide:bis(acrylamide)) electrophoresis using a model 491 prep cell (Bio-Rad). The prep cell was run at 6 W, and after 1 h, 1.2 mL fractions were collected in a mixture of 10 mM Tris-HCl pH 8.0, 1 mM EDTA, and 1 mM DTT at a flow rate 0.3 mL/min. Purification was checked by 4% acrylamide native electrophoresis, and fractions containing cross-linked nucleosomes were separately concentrated by centrifugation using 100 K Amicon Ultra filters (Millipore). After concentration, samples were dialyzed against HE buffer (20 mM HEPES pH 7.5, 1 mM EDTA, and 1 mM DTT) and stored at 4 °C.

### Atomic Force Microscopy Imaging in Air

Prior to any dynamic studies, the quality and purity of all the samples—chromatosomes, nucleosomes, hexasomes, and tetrasomes—were evaluated by AFM in air. In this case, we imaged formaldehyde cross-linked samples which were diluted in 10 mM MOPS pH 7.0 and 2 mM MgCl_2_ to concentrations ranging between 2 and 4 nM. Three microliters of the solution was deposited onto freshly cleaved bare mica V1 (Ted Pella Inc.) and incubated for 2 min, gently rinsed with Milli-Q water, and dried under a stream of nitrogen. Tetrasomes using the 100W50 template were visualized by deposition on polylysine mica (0.1% v/v) and rinsed and dried as above. AFM measurements were performed with a Multimode AFM Nanoscope 8 (Bruker Co.). The samples were imaged in tapping mode; the silicon cantilevers (Nanosensors) were excited at their resonance frequency (280–350 kHz) with free amplitudes of 2–10 nm. The image amplitude (set point *A*_s_) and free amplitude (*A*_0_) ratio (*A*_s_/*A*_0_) were kept at 0.8, and the scan rate was kept at 2 Hz (∼0.006 fps). All samples were imaged at room temperature in air, at a relative humidity of 30%.

### High-Speed AFM

To characterize the nucleosomes dynamics in 2D, individual molecules were observed in buffer using the Ando-model HS-AFM-Ando (Research Institute of Biomolecule Metrology). Nucleosome samples were serially diluted up to 900-fold and incubated on ice for 30 to 90 min at a concentration of 1.5–3.5 nM, and then they were deposited onto freshly cleaved mica for 2 min in buffer A (5 mM MOPS pH 7.0, 80 mM KCl, 20 mM NaCl, 0.5 mM EGTA, 2 mM EDTA, 0.5 mM Spermidine, 0.2 mM spermine, 5 mM Na(C_3_H_7_COO), and 1 mM DTT). The surface was rinsed to remove unbound species and imaged in 10-fold-diluted buffer A by HS-AFM at one or two frames per second (fps). Data acquisition was initiated by first surveying the surface to locate well-separated molecules, enabling the observation of one molecule at a time and thereby minimizing unwanted interactions with other molecules. A typical acquisition session lasted a maximum of 2 h of continuous inspection of the same mica. We made fresh dilutions and depositions every 3 h. The samples were scanned in 10-fold-diluted buffer A using tapping mode at room temperature. The deflection of a micrometer-sized cantilever (USC-F1.2-K0.15-10, Nanoworld, spring constant ∼0.1 N/m, resonance frequency 500–600 kHz in liquid) was detected using an optical beam detector. The *A*_0_ was set to ∼2 nm, and the *A*_s_/*A*_0_ ratio was kept high to achieve the highest resolution at the lowest force possible.

### Data Analysis

#### Image Processing

Raw static AFM images acquired in air were flattened and leveled using Gwyddion 2.59.^64^ Individual frames from HS-AFM movies were preprocessed using customized algorithms written in Igor Pro (Wave Metrics Inc. Oregon). The noise was reduced by Gaussian filtering followed by a flattening filter, and then the entire molecule was tracked using a 2D correlation method to reduce lateral drift.^65^ To extract quantitative information from each frame, masks of the DNA and the NCP were generated manually in Gwyddion to identify the two nucleosomal components (NCP and DNA) separately. Approximately 10% of the frames were manually segmented in Gwyddion into three classes: background (89% of the pixels), nucleosome core particle (NCP) (3%), and DNA (7%). These hand-generated masks were used to train a supervised neural network classifier (implemented using PyTorch), which took a 11 × 11 median-subtracted subimage as input (for pixels at the edge of the image, the image was padded symmetrically) and aimed to predict class logits of the central pixel. The neural network had a single fully connected hidden layer of 121 neurons using ReLU activation (Figure SI 2B) and was trained using standard backpropagation. The subimage size and network architecture were optimized empirically. After training, 91% of the background pixels, 97% of the NCP pixels, and 88% of the DNA pixels were correctly labeled by the classifier (Table SI 3; e.g., actual background pixels represent 89.2% of all pixels and actual background pixels that the classifier correctly predicted as background amount to 81.3% of all pixels; the correct labeling rate for background pixels is thus 81.3%/89.2% = 91.1%). Visual inspection of the incorrectly labeled pixels showed that many of the errors arose from pixels at the border between two classes (e.g., at the edge of a nucleosome) and that could therefore reasonably be labeled with either class; thus, the practical performance of the classifier was even higher.

The trained classifier was then used to segment all remaining frames. The largest connected component of pixels labeled by the neural net as NCP was considered to be the main particle. Although we collected many suitable molecules, we restricted our analyses to isolated nucleosomes that interact minimally with neighboring molecules to minimize misassignments and errors in the unsupervised segmentation. Each frame from a given movie was manually inspected and corrected, when required, to further ensure the accuracy of the NCP and DNA assignments.

#### Molecular Measurements

Having properly segmented molecules, we proceeded to measure the DNA arms’ length (for molecules imaged in air), the DNA angle formed at the entry/exit site of the nucleosomes, and the histone core’s volume for molecules imaged in buffer. To measure the DNA contour length (DNA arms’ length), isolated molecules were cropped (∼30 molecules/micrograph) from micrographs of 500 × 500 nm^2^ corresponding to 100W50 tetrasomes imaged in air. The images were flattened, and masks of each tetrasome were manually extracted from each molecule using Gwyddion. The binary masks were used to obtain an initial DNA trace using the bwskel skeletonization algorithm in Matlab (Mathworks Ltd. Natick, MA), and such traces were smoothed and refined using active contour models using the scikit-image library in Python. Due to the intrinsic noise of the images, the DNA traces missed 2 pixels (∼3.8 nm) at each end. Thus, the measured full length of the DNA was ∼90 nm instead of the expected 98 nm (297 bp × 0.33 nm/bp). The DNA skeletons were smoothed to eliminate artifactual kinks, the center of mass of the histone tetramer was determined, and the length from the center of mass of the tetramer to the end of each DNA skeleton was computed using home-written Python codes. Faulty DNA tracing or tetramer assignments were manually removed from the data set. DNA lengths of the short and long DNA arms were used for further analysis.

Measuring the length of nucleosome DNA arms in HS-AFM images presented exceptional challenges due to their unpredicted movement and intermittent detachment, which caused a loss of information in many of the frames. Nonetheless, we were able to extract a few frames from Movies SI 2 and SI 4 in which the DNA became fully visible following a dimer eviction event. This allowed us to measure DNA length, using the method described above, and evaluate changes in the DNA arm ratio (*R*_DNA_) resulting from DNA unwrapping and subsequent histone dimer release.

The volume of dried molecules was measured using the Laplacian background basis from Gwyddion’s grain measurement module.^64^ The values obtained are in reasonable agreement with those reported in the literature,^13,28^ although they tend to be underestimated due to molecular dehydration and inaccuracy in the background under the particle approximation. Conversely, the volumes of molecules imaged in liquid are high due to hydration and the way of determining the background height under the nucleosome. In this case, the background was estimated as follows: pixels which were predicted to be nonempty (i.e., NCP or DNA) were masked out and refilled using OpenCV’s INPAINT_NS (Navier–Stokes) inpainting algorithm.

The angles formed by the DNA at the entry–exit sites of each molecule were also determined. First, among the pixels classified as “DNA” by the neural network classifier, those that are part of a connected component smaller than half the largest connected component of “DNA” pixels were discarded (thus throwing away small noisy fluctuations by the classifier). The residual pixels were split into the two DNA arms as follows: the area defined by the pixels classified as “DNA” or “nucleosome” were skeletonized, and a metric which strongly favors motion along the skeleton was defined. Then, the end of the first DNA arm was identified as the farthest DNA pixel in this skeleton from the centroid of the pixels classified as nucleosomes, and the extremity of the second arm was determined as the farthest DNA pixel in this skeleton from both the nucleosome centroid and the first arm extremity. The remaining DNA pixels were then assigned to either arm using a random-walker-type algorithm, with parameters chosen so that the walkers meet, on average, at the nucleosome center. This procedure separated the two nucleosome arms, as shown in Figure 1B (ii). Finally, the coordinates of the pixels in each DNA arm were expressed in polar coordinates, taking the nucleosome center as the origin. These pixels define a θ(*r*) relation, which was smoothed using the scipy.interpolate.UnivariateSpline method; the smoothed θ(*r*) value at the border between the nucleosome and the DNA arm defines the orientation θ_1_ or θ_2_ (relative to an arbitrary origin) of the arm at the entry or exit site. The method we employed was suitable for assessing the general angular changes in the DNA arms and identifying which arm angles were affected during histone dimer ejection. We note that our methodology has limitations when we observed (i) the entire particle displaced within the frame, (ii) unexpected collisions with neighboring molecules which caused changes of orientation of the molecules, and (iii) transient desorption of the DNA arms. To ensure the accuracy and reliability of our reporting on angular changes at specific nucleosome sites, we limited the analysis of other nucleosomes to frames captured in the vicinity of the dimer ejection event (approximately 8 frames before and after the ejection). Additionally, we enhanced the precision of our measurements by conducting manual verification and correction of any miscalculated angles using Gwyddion.

## Supporting Information Available

Characterization of the assembly and purification of nucleosomes, PANS, and chromatosomes by gel electrophoresis and effect of high-speed scanning on nucleosome molecular dynamics and disassembly (PDF)
Nucleosome and DNA segmentation (MPG)
Dynamic disassembly of a nucleosome in real time (MPG)
Two different mechanisms of nucleosomal disassembly (MPG)
Dynamic disassembly of a hexasome in real time (MPG)
Dynamic disassembly of a tetrasome in real time (MPG)
Real-time dynamics of a de novo tetrasome (MP4)
Dynamic disassembly of a chromatosome in real time (MPG)
Real-time dynamics of a nucleosome cross-linked with formaldehyde (MP4)
Real-time dynamics of a nucleosome cross-linked with DMS (MP4)
Transparent Peer Review report available (PDF)

## Supplementary Material

oc3c00735_si_005.pdf

oc3c00735_si_006.mpg

oc3c00735_si_007.mpg

oc3c00735_si_008.mpg

oc3c00735_si_009.mpg

oc3c00735_si_010.mpg

oc3c00735_si_011.mp4

oc3c00735_si_012.mpg
