# Supplementary material for: Real-Time Multistep Asymmetrical Disassembly of Nucleosomes and Chromatosomes Visualized by High-Speed Atomic Force Microscopy
Source: ACS Cent Sci. 2023 Dec 22;10(1):122–37. doi: 10.1021/acscentsci.3c00735 (PMC10823521; doi:10.1021/acscentsci.3c00735)
Supplement: Supplementary file 1 — oc3c00735_si_005.pdf [file oc3c00735_si_005.pdf]

Real-Time Multistep Asymmetrical Disassembly of Nucleosomes and Chromatosomes Visualized by High-Speed Atomic Force Microscopy

Bibiana Onoa,[*,†,‡,§,#] **César Díaz-Celis,** [†,‡,§] **Cristhian Cañari-Chumpitaz,**[†,‡,§,∇] **Antony Lee,**[∕∕,+] and **Carlos Bustamante** [*,†,‡, §,⊥]

†Jason L. Choy Laboratory of Single-Molecule Biophysics, University of California, Berkeley, California 94720, United States

‡Howard Hughes Medical Institute, University of California, Berkeley, California 94720, United States

§ California Institute for Quantitative Biosciences, QB3, University of California, Berkeley, California 94720, United States

∕∕Laboratoire Photonique Numerique et Nanosciences, LP2N UMR 5298, Universite de Bordeaux, Institut d'Optique, CNRS, F-33400Talence, France

⊥Kavli Energy Nanoscience Institute, University of California, Berkeley, California94720, United States

*Email: bibianaonoa@berkeley.edu.

*Email: carlosjbustamante2@gmail.com.

Characterization of the assembly and purification of nucleosomes, PANS, and chromatosomes by gel electrophoresis

Nucleosomes and hexasomes purification

The process of assembling human nucleosomes on the 601 NPC via salt dialysis leads to the formation of both nucleosomes and hexasomes, as evident from the results of 4% acrylamide native electrophoresis (Fig. SI 1A, left panel). The intensity of the gel bands indicates that the nucleosome assembly reaction comprises 55%, 23%, and 22% of nucleosomes, hexasomes, and free DNA template (447 bp), respectively. To isolate hexasomes and nucleosomes, and to reduce the amount of free DNA, the nucleosome assembly reaction was purified using 4% acrylamide preparative electrophoresis (Fig. SI 1A), which separated the free DNA (447 bp DNA template, first to elute in fractions number 5 and 6) from hexasomes (fractions 8, 9, and 10) and nucleosomes (fractions number 34 to 42) (Fig. SI 1A, middle panel). Analysis of the band intensity reveals that the hexasome fraction number 9 contains 85% of hexasomes and 15% of DNA. Meanwhile, nucleosome fraction number 38 contains a mix of 78% nucleosomes, 16% hexasomes, and 6% DNA. Fractions containing hexasomes (H) and nucleosomes (N) were combined, concentrated (~90X for hexasomes and ~250X for nucleosomes), and dialyzed against 20 mM HEPES pH 7.5, 1 mM EDTA, and 1 mM DTT (Fig. SI 1A, right panel). The analysis of the native gel (4% acrylamide; ~40 ng DNA for each sample) shows that the hexasome sample is practically nucleosome free and contains 75% of hexasomes and 25% free DNA. The purified nucleosome contains 71% nucleosomes, 21% hexasomes, and 8% free DNA. This suggests that the purification process enriched the proportion of hexasomes and nucleosomes in each purified species compared to the input (I). However, an increase in free DNA and hexasomes in the nucleosome sample compared to fraction 39, as well as an increase in free DNA in the hexasome sample compared to fraction 9, suggests that hexasomes and nucleosomes disassemble during their fractionation through the concentration process. The final concentration of nucleosomes and hexasomes was ~1.4 µM and 0.25 µM, respectively. These samples were diluted to 1.5 nM (or ~170X for hexasomes and 900X for nucleosomes) for HSAFM experiments.

De novo tetrasomes purification

Human 601 tetrasomes were assembled using pure $(H3{\cdot}H4)_2$ tetramers and a 297 bp DNA template (same procedure was followed for tetrasomes assembled with a 447 bp DNA template). This assembly process resulted in two main bands (Fig. 1B, left panel). The upper band correspond to the tetrasome, while the lower band corresponds to octameric-like particles formed by two $(H3{\cdot}H4)_2$ as previously described[1, 2]. In this assembly reaction, the species composition was 34% tetrasomes, 32% octasome-like particles, and 34% free DNA. The human tetrasome was purified using ultracentrifigation with a 5-20% sucrose gradient (Fig. SI 1B, middle panel). Fractions 12 and 13, which contained the purified tetrasome (T) at a higher ratio compared to free DNA, were combined and concentrated (Fig SI 1B, right panel). The band intensities reveal that the purified tetrasome sample comprises 52% tetrasomes, 2% octasome-like particles, and 46% DNA. The higher proportion of free DNA in the purified tetrasomes, as opposed to the unpurified ones, suggests that our ultracentrifugation purification method promotes their disassembly. It's important to note that free DNA was excluded from our AFM experiments.

Chromatosome purification

The addition of linker histone H1 to nucleosomes in a 1:1 ratio result in the formation of a distinct band, slightly shifted from the nucleosome band towards the upper region of the native gel (Fig. SI 1C, left panel). This band corresponds to the binding of H1 to the nucleosome, resulting in the formation of a chromatosome. Further increasing the concentration of H1 leads to the complete assembly of chromatosomes. Notably, the increase in the amount of H1 gives rise to several intermediate bands while concurrently reducing the presence of free DNA and hexasomes. This phenomenon suggests that these intermediate species are the result of H1 binding to DNA and to hexasomes. For example, at a ratio of 1:2 nucleosome:H1, a new band appears above the hexasome, indicating the presence of the H1-hexasome complex. Chromatosomes formed at a 1:4 nucleosome:H1 ratio were purified using a 5-30% sucrose gradient (Fig. SI 1C, middle panel). The H1-hexasome complex is primarily in fraction number 14 and corresponds to the second bigger complex after the cromathosome. Fractions 17 to 20 containing the chromatosome were combined and concentrated (Fig. SI 1C, right panel). Our purification method reduces the number of intermediates and free DNA in the chromatosomes. This significant reduction of free DNA in our purified chromatosomes in comparison to purified nucleosomes, hexasomes, and tetrasomes underscores the role of H1 in stabilizing nucleosomes.

Effect of high-speed scanning on nucleosome molecular dynamics and disassembly

Since native nucleosomes disassemble during the AFM observation without additional input of energy, questions then arise: is the dissociation induced by the tip-molecule interactions, and/or by interactions between the surface and the molecule? What is the effect of the scanning rate? Certainly, all these experimental factors can play a role in the observed dynamics, however, there is compelling experimental evidence indicating that they do not govern it. For example, when scanning simultaneously several nucleosomes in close proximity, different survival times and disassembly mechanisms are observed for each one of them (Fig SI 3B). As stated in the manuscript, tetrasomes and chromatosomes deposited and scanned under similar experimental conditions than nucleosomes display lifetimes two to five-fold longer than the latter. Different types of nucleosomes (e.g., CENP-A with different histone composition, different elastic properties, and wrapping mode) also display longer lifetimes than those of canonical nucleosomes and follows different pathways[3, 4]. Scanning two-fold slower does not increase the nucleosome's lifetime (Fig. SI 11). By scanning the same area on and off (lifting the probe for several minutes between scans) Katan et al. observed that nucleosomes dissociate even when they do not interact with the AFM tip[5]. Shlyakhtenko et al. compared a large field of view—crowded with nucleosomes at time zero—before and after scanning a smaller section continuously, they detected nucleosomal dissociation in both heavily scanned as well as lightly scanned areas [6]. Furthermore, Miyagi et al. estimated the force/impulse and energy transferred by the tip to the molecules during an oscillation cycle under standard HS-AFM scanning conditions (see Methods) and concluded that their magnitude could not account for the level of nucleosome dynamics observed, and by extension, for the massive molecular dissociation effect detected[7]. Recently, Melters et al. reported that HS-AFM imaging is free of tip-induced artifacts since they did not observe any bias in the fast and slow scan axes on single molecule tracking trajectory distributions—step angle, step size, diffusion constant—of nucleosomes or nucleosome arrays[4].

To minimize significant changes of the forces between the tip and the molecules due to water evaporation and the consequent change in ionic strength of the scanning media, we also kept our scanning buffer volume as constant as possible. Lastly, to uncouple the structural contributions of nucleosomes on their disassembly from the effects of the AFM tip-sample interactions, we imaged cross-linked nucleosomes to prevent DNA unwrapping and NCP rearrangement. We employed formaldehyde to prevent both DNA unwrapping and NCP rearrangement. Additionally, to specifically curtail histone mobility, we employed DMS. In both scenarios, the nucleosomes remained intact throughout the observation, even when subjected to increased tip forces applied to the sample (Figs. SI 8 and SI 10, and Movies SI 8 and SI 9). Therefore, while we do not rule out that the intrinsic AFM scanning conditions might influence the nucleosome's dynamics and disassembly, they are not fully responsible for them.

An unavoidable requirement for AFM scanning in solution is to achieve a stable sample-surface interaction without abolishing the molecule's intrinsic dynamics. To meet this challenging condition, the experimental choice is to balance the electrostatic forces between molecule and substrate. In most studies found in the literature, the nucleosomes are immobilized on positively charged substrates of different nature. Conversely, in our experimental strategy, the surface is not pre-treated, but the nucleosomes are suspended in a cationic solution (i.e., monovalent ions and poly-valent amines) that shields the strong repulsive interaction between sample and substrate[9], allowing its adsorption on a mainly negatively charged surface. Indeed, nucleosomes deposited on unmodified mica do not disassemble upon extensive HS-AFM scanning (> 5 min) when DNA unwrapping is prevented[10].

DNA opening angles (formed between the DNA arms and the center of mass of the NCP) have been measured on AFM images of nucleosomes to determine the degree of DNA nucleosomal unwrapping. However, we notice that these angular values are convoluted with the orientation adopted by the complex during its adsorption onto the surface. To illustrate this point, we created surface maps of the nucleosome, PANS, and chromatosome using coordinates reported in the literature[11, 12]. We arbitrarily define an initial orientation for each molecular map as zero angle (e.g., a top view of the molecular surface map parallel to the surface to mimic a random orientation at time zero). We then systematically rotated each surface map around the X-axis (because it is the fast-scanning axis) by 15° until reaching a full rotation (180°) (Fig. SI 12). This in-silico rotational exercise of static molecular maps allowed us to unveil that the apparent DNA opening angle can change broadly without invoking DNA unwrapping. For example, figure SI 12 illustrates that the DNA opening angle of a tetrasome is significantly different when the molecule is observed at zero degrees compared to 75 degrees. Likewise, the morphology of the histone core appears different depending on its orientation on the surface (Fig. SI 12). Lastly, the comparison of the histone core morphology evolution from our AFM recordings (Figs. 1A, 1C, 2A, 2C, 4A, 4C, SI 3 and SI 5 and Movies SI 2 – SI 9) with those presented in figure SI 12 also permit us to determine that the molecules did not experience major rotation (>45°) during our observations since we did not see equivalent morphological transitions.

Table SI 1. Length of the DNA arms measured during the disassembly of a nucleosome and a hexasome

**Molecule**	**Expected DNA arm ratio (R_{DNA})**	**Measured Total length (nm)**	**Measured long DNA arm (nm)**	**Measured short DNA arm (nm)**	**Measured DNA arm ratio (R_{DNA})**
Nucleosome Fig. 1A	~2:1	115	70	46	1.6:1
Hexasome Fig. 1A	~1.5:1	126	74	52	1.4:1
Tetrasome Fig. 1A	~2:1	130	73	57	1.3:1
Hexasome Fig. 2A	~1.5:1	127	77	55	1.3:1
Tetrasome Fig. 2A	~2:1	137	70	67	1.05:1

Table SI 2. Dynamic volume variation of different species measured in real-time HS-AFM

	Mean (nm^3)	Standard Deviation (nm^3)	FWHM
Chromatosomes	673.7	155.8	111
Nucleosomes	594.8	116.3	70
Hexasomes	470.2	113.6	133
Tetrasomes	351.7	74.5	91.8

FWHM: Full width at half maximum

Table SI 3. True and predicted labels of segmented images by the supervised neural network classifier

		**Predicted label**		
		Background: 81.7%	DNA: 12.3%	NCP: 5.4%
**True label**	Background: 89.2%	81.3%	6.4%	1.5%
	DNA: 7.2%	0.4%	6.4%	0.4%
	NCP: 3.5%	0.0%	0.1%	3.5%

Figure captions

Figure SI-1. Assembly and purification of human nucleosomes, hexasomes, tetrasomes, and chromatosomes. (A) The assembly of human histone octamers on Widom 601 NCP (input (I)) results in 55% nucleosomes (N), 23% hexasomes (H), and 22% free DNA (left panel). After purification by electrophoresis (middle panel), enriched fractions with N or H were combined and concentrated. Purified H samples contain 75% hexasomes and 25% free DNA, while purified N samples comprise 71% nucleosomes, 21% hexasomes, and 8% free DNA (right panel). **(B)** The assembly of human $(H3{\cdot}H4)_2$ tetramers on 601 NCP (I) yields 34% tetrasomes, 32% octasome-like particles, and 34% free DNA (left panel). Tetrasomes from the input were purified through ultracentrifugation using a 5-20% sucrose gradient (middle panel). Fractions 12 and 13 were combined and concentrated, resulting in 52% tetrasomes, 2% octasome-like particles, and 46% free DNA (right panel). **(C)** Addition of the linker histone H1.0 to nucleosomes leads to the assembly of chromatosomes as well as H1-hexasomes (left panel). Chromatosomes formed at a ratio of 1:4 (nucleosome:H1) were purified using a 5-30% sucrose gradient (middle panel). Fractions 17 to 20 were combined and concentrated (right panel).

Figure SI-2. A diverse mixture of species coexists on the surface before conducting targeted AFM scanning of nucleosomes and algorithm development for image segmentation (**A**) Representative AFM images showcasing different sample depositions conducted as part of the pre-selection process to identify suitable nucleosomes for dynamic observation. (**B**) Architecture of the supervised neural network classifier algorithm.

Figure SI-3. Nucleosome disassembly duration is stochastic. (A) Two nucleosomes in close proximity displaying different morphology, orientation and NCP volume (top 630 nm^3 and bottom 867 nm^3), the double headed arrow lines specify the short DNA arm indicating that both molecules are intact nucleosomes. **(B)** Disassembly time lapse of the molecules depicted in A. Despite the large fluctuations of the DNA linker of the top nucleosome, it remains intact for a longer time (43 s) than the bottom nucleosome (39 s). Both nucleosomes disassembled into tetrasomes by the end of the observation. Note that the ejected histone dimer of the bottom nucleosome glides away following the DNA linker as a track (yellow arrows). The red crosses highlight the invasion of DNA from a neighboring molecule. Z-color-map from 0 to 6 nm.

Figure SI-4. Asymmetry in the heterodimer ejection observed in several nucleosomes (A) Aligned volume changes during the transition from nucleosome to hexasome for five different nucleosomes with the corresponding angular changes at the entry (long DNA arm) and exit (short DNA arm) site of the NCP **(B)** Equivalent volume and angular changes monitored during the transition from hexasome to tetrasome for the same five molecules.

Figure SI-5. Comparison of DNA length ratios of tetrasomes of different origin (A) Excerpts from movies recording the disassembly of a nucleosome (Fig. 1A and Movie SI 2, top row) or a hexasome (Fig. 2A and Movie SI 4, bottom row) used to measure their DNA arm's length and to determine how their R_{DNA} (length ratio of the long and short DNA arms) changes during the process (Table SI 1). **(B)** Schematic representation of a nucleosome, hexasome, and tetrasome with their respective expected DNA length when measured using the methodology described in Methods. The expected diameter (Ø) of the histone cores (green quasi-spherical shapes) was estimated from the actual AFM images obtained during the disassembly process. **(C)** Air tapping mode AFM micrograph of de novo tetrasomes on the long DNA template (200W100) scanned at 0.006 fps on poly-lysine coated mica, note the high variability of R_{DNA}. Z-color-map from 0 to 6 nm.

Figure SI-6. **AFM characterization of chromatosomes. (A)** AFM micrograph of cross-linked nucleosomes scanned in air at ~0.006 fps (top panel) and their calculated nucleosome core particle (NCP) volume showing a normal distribution (bottom panel). NCP volumes were measure using the Laplacian background basis from Gwyddion's grain measurement module[13]. μ = mean and σ = SD. **(B)** AFM micrograph of cross-linked chromatosomes and their NCP volumes normally distributed. White circles highlight the teardrop morphology. The goodness of the Gaussian fitting (black lines) was determined by the Akaike information criterion. **(C)** Expected volume comparison of surface maps of a nucleosome (PBD 6ESF)[14] and a chromatosome (PBD 4QLC)[15] at atomic resolution (top panels) or at a resolution comparable to that obtained by AFM (bottom panels)[16]. (**D**) Comparison of the experimental AFM chromatosome in Fig. 4A and its equivalent atomic surface map as a visual guide to determine its initial orientation on the surface[17]. A yellow arrow points out the linker histone H1.

Figure SI-7. Volume distributions and lifetime of the first ejection event of chromatosomes, nucleosomes, and Hexasomes. (A) Schematic representation to illustrate the measured time or volume for the different types of molecules. **(B)** Kernel density estimator of the real-time distributions of volumes recorded during the disassembly of several chromatosomes (N = 8; 522 frames) nucleosomes (N = 9; 376 frames), hexasomes (N = 13; 330 frames), and tetrasomes (N = 3; 384 frames). **(C)** Cumulative distribution function illustrates the time elapsed before the first heterodimer dissociation event for chromatosomes (N = 8), nucleosomes (N = 9), and purified hexasomes (N = 13).

Figure SI-8. Inhibition of DNA unwrapping abolishes nucleosome disassembly (A) Time-lapse of a cross-linked nucleosome with formaldehyde **(B)** volume evolution trajectory of molecule in (A) (RMS = 973.7). False Z-color-map from 0 to 6 nm. Purple ticks and numbers in the plots show the time point displayed in the images.

Figure SI 9. DMS cross-linking and purification of nucleosomes (A) Nucleosomes were assembled by dialysis, cross-linked with DMS, and their stability evaluated by native 4% acrylamide electrophoresis. The cross-linked assembly reaction includes free DNA, cross-linked hexasomes (X-hexasomes), cross-linked nucleosomes (X-nucleosomes), and cross-linked species and/or aggregates of high molecular weight. X-nucleosomes and X-hexasomes migrate to a position like that of native nucleosomes (Fig. SI 1A). **(B)** The degree of the histone cross-linking was assessed by electrophoresis in an SDS-PAGE gel. While non-cross-linked histones migrate as three bands of ~15 kDa, cross-linked histone octamer (~108 kDa) migrates mainly as a single band (between 100 kDa and 130 kDa) along with partially cross-linked histones. The intensity of the bands indicates that the cross-linked nucleosomes represent ~95% of the species. **(C)** The input was purified by electrophoresis. X-hexasome eluted in fractions 10 - 12, and X-nucleosomes eluted in fractions 25 to 29. **(D)** Fractions containing X-hexasomes and X-nucleosomes were combined and concentrated to ~1 μM. The low amount of hexasomes and free DNA in the purified X-nucleosome sample indicates that DMS effectively prevents nucleosomal disassembly.

Figure SI-10. Stabilization of the nucleosome core effectively inhibits nucleosome disassembly (A) Time excerpts of a cross-linked nucleosome with DMS **(B)** Corresponding volume trajectories (RMS = 1099.2). Yellow arrows denote streaks, caused by transient amplitude setpoint decreases accompanied by an increase in force. Yellow rectangles in the micrographs and purple lines in the volume trace indicate the rapid recovery of NCP morphology and volume within a second after increasing the tip force during scanning. The black arrow signifies the time when the tip was momentarily lifted from and re-engaged with the same nucleosome. The false-color Z-map spans from 0 to 6 nm.

Figure SI-11. **Nucleosome disassembly pathway is independent of AFM scan rate.** Nucleosome's volume as a function of time follows similar trajectories to those shown in Fig. 1C when the scanning rate is slower (1 frame per second (fps))

Figure SI-12. Apparent molecular morphology depends on the 2D orientation adopted during deposition onto the substrate. Top view juxtaposition of molecular surface maps of a chromatosome (PDB: 5NL0)[11], nucleosome (PDB:1KX5)[18] and PNAS particles (PDB from Rychkov et al.)[12] observed at different orientations (X-rotation: step 15°)[17].

Movie captions

Movie SI-1. Nucleosome and DNA segmentation. Example of a nucleosome (Fig. 1A) with automatic segmentation and tracing by the neural classifier algorithm. Nucleosome core particle (NCP) is depicted by blue curves while the DNA arms by red curves; each linker is traced by a green line and by an orange line.

Movie SI-2. Dynamic disassembly of a nucleosome in real-time. Disassembly of the nucleosome shown in Fig. 1A scanned at 2 fps and played at 4x. The Z-color-map (0 to 6 nm) displays changes in color distribution—but not the values—due to changes in the height of the structures of each entire frame. Note that when the maximum height of the particles decreases due to molecular dissociation events, the Z-colors encoding higher height values do not disappear but are diagonally striped indicating that those values are no longer detected in the frame(s).

Movie SI-3. Two different mechanisms of nucleosomal disassembly. Two nucleosomes (Fig. SI 3) scanned simultaneously at 2 fps displaying different rates and mechanism of dynamical disassembly. Movie speeded up 4x. Z-color-map 0 to 6 nm.

Movie SI-4. Dynamic disassembly of a hexasome in real-time. Disassembly of the hexasome shown in Fig. 2A scanned at 2 fps and played at 5x. Z-color-map 0 to 5.5 nm.

Movie SI-5. Dynamic disassembly of a tetrasome in real-time. Disassembly of the tetrasome shown in Fig. 2C scanned at 2 fps and played at 5x. Z-color-map 0 to 5.5 nm.

Movie SI-6. Real-time dynamics of a de novo tetrasome. Dynamics of a tetrasome with the DNA arms in a close configuration scanned at 2 fps and played at 5x. Z-color-map 0 to 5.5 nm.

Movie SI-7. Dynamic disassembly of a chromatosome in real-time. Disassembly of the chromatosome shown in Fig. 4A scanned at 1fps and played at 5x. Z-color-map 0 to 6 nm.

Movie SI-8. Real-time dynamics of a nucleosome cross-linked with formaldehyde. Dynamics of the nucleosome cross-linked with formaldehyde shown in Fig. SI 8 scanned at 2 fps and played at 5x. Z-color-map 0 to 6 nm.

Movie SI-9. Real-time dynamics of a nucleosome cross-linked with DMS. Dynamics of the nucleosome cross-linked with DMS shown in Fig. SI 10 scanned at 2 fps and played at 5x. Z-color-map 0 to 6 nm.

Bibliography

(1) Diaz-Celis, C.; Canari-Chumpitaz, C.; Sosa, R. P.; Castillo, J. P.; Zhang, M.; Cheng, E.; Chen, A. Q.; Vien, M.; Kim, J.; Onoa, B.; et al. Assignment of structural transitions during mechanical unwrapping of nucleosomes and their disassembly products. *Proc Natl Acad Sci U S A* **2022**, *119* (33), e2206513119. DOI: 10.1073/pnas.2206513119 From NLM Medline.
(2) Zou, T.; Hashiya, F.; Wei, Y.; Yu, Z.; Pandian, G. N.; Sugiyama, H. Direct Observation of H3-H4 Octasome by High-Speed AFM. *Chemistry* **2018**, *24* (60), 15998-16002. DOI: 10.1002/chem.201804010.
(3) Stumme-Diers, M. P.; Banerjee, S.; Hashemi, M.; Sun, Z.; Lyubchenko, Y. L. Nanoscale dynamics of centromere nucleosomes and the critical roles of CENP-A. *Nucleic Acids Res* **2018**, *46* (1), 94-103. DOI: 10.1093/nar/gkx933.
(4) Melters, D. P., Neuman, K. C., Rakshit T., Dalal Y. . Single Molecule Analysis of CENP-A Chromatin by High-Speed Atomic Force Microscopy. *BioRxiv* **2023**. DOI: 10.1101/2022.01.04.474986.
(5) Katan, A. J.; Vlijm, R.; Lusser, A.; Dekker, C. Dynamics of nucleosomal structures measured by high-speed atomic force microscopy. *Small* **2015**, *11* (8), 976-984. DOI: 10.1002/smll.201401318.
(6) Shlyakhtenko, L. S.; Lushnikov, A. Y.; Lyubchenko, Y. L. Dynamics of nucleosomes revealed by time-lapse atomic force microscopy. *Biochemistry* **2009**, *48* (33), 7842-7848. DOI: 10.1021/bi900977t.
(7) Miyagi, A.; Ando, T.; Lyubchenko, Y. L. Dynamics of nucleosomes assessed with time-lapse high-speed atomic force microscopy. *Biochemistry* **2011**, *50* (37), 7901-7908. DOI: 10.1021/bi200946z.
(8) Casuso, I.; Scheuring, S. Automated setpoint adjustment for biological contact mode atomic force microscopy imaging. *Nanotechnology* **2010**, *21* (3), 035104. DOI: 10.1088/0957-4484/21/3/035104 From NLM Medline.
(9) Gebala, M.; Johnson, S. L.; Narlikar, G. J.; Herschlag, D. Ion counting demonstrates a high electrostatic field generated by the nucleosome. *Elife* **2019**, *8*. DOI: 10.7554/eLife.44993.
(10) Feng, Y.; Hashiya, F.; Hidaka, K.; Sugiyama, H.; Endo, M. Direct Observation of Dynamic Interactions between Orientation-Controlled Nucleosomes in a DNA Origami Frame. *Chemistry* **2020**, *26* (66), 15282-15289. DOI: 10.1002/chem.202003071 From NLM Medline.
(11) Bednar, J.; Garcia-Saez, I.; Boopathi, R.; Cutter, A. R.; Papai, G.; Reymer, A.; Syed, S. H.; Lone, I. N.; Tonchev, O.; Crucifix, C.; et al. Structure and Dynamics of a 197 bp Nucleosome in Complex with Linker Histone H1. *Mol Cell* **2017**, *66* (5), 729. DOI: 10.1016/j.molcel.2017.05.018.
(12) Rychkov, G. N.; Ilatovskiy, A. V.; Nazarov, I. B.; Shvetsov, A. V.; Lebedev, D. V.; Konev, A. Y.; Isaev-Ivanov, V. V.; Onufriev, A. V. Partially Assembled Nucleosome Structures at Atomic Detail. *Biophys J* **2017**, *112* (3), 460-472. DOI: 10.1016/j.bpj.2016.10.041.
(13) Necas, D.; Klapetek, P. Gwyddion: an open-source software for SPM data analysis. *Cent Eur J Phys* **2012**, *10* (1), 181-188. DOI: 10.2478/s11534-011-0096-2.
(14) Bilokapic, S.; Strauss, M.; Halic, M. Histone octamer rearranges to adapt to DNA unwrapping. *Nat Struct Mol Biol* **2018**, *25* (1), 101-108. DOI: 10.1038/s41594-017-0005-5.
(15) Zhou, B. R.; Jiang, J.; Feng, H.; Ghirlando, R.; Xiao, T. S.; Bai, Y. Structural Mechanisms of Nucleosome Recognition by Linker Histones. *Mol Cell* **2015**, *59* (4), 628-638. DOI: 10.1016/j.molcel.2015.06.025.
(16) Voss, N. R.; Gerstein, M. 3V: cavity, channel and cleft volume calculator and extractor. *Nucleic Acids Res* **2010**, *38* (Web Server issue), W555-562. DOI: 10.1093/nar/gkq395.
(17) Amyot, R.; Marchesi, A.; Franz, C. M.; Casuso, I.; Flechsig, H. Simulation atomic force microscopy for atomic reconstruction of biomolecular structures from resolution-limited experimental images. *PLoS Comput Biol* **2022**, *18* (3), e1009970. DOI: 10.1371/journal.pcbi.1009970 From NLM Medline.
(18) Gansen, A.; Valeri, A.; Hauger, F.; Felekyan, S.; Kalinin, S.; Toth, K.; Langowski, J.; Seidel, C. A. Nucleosome disassembly intermediates characterized by single-molecule FRET. *Proc Natl Acad Sci U S A* **2009**, *106* (36), 15308-15313. DOI: 10.1073/pnas.0903005106.

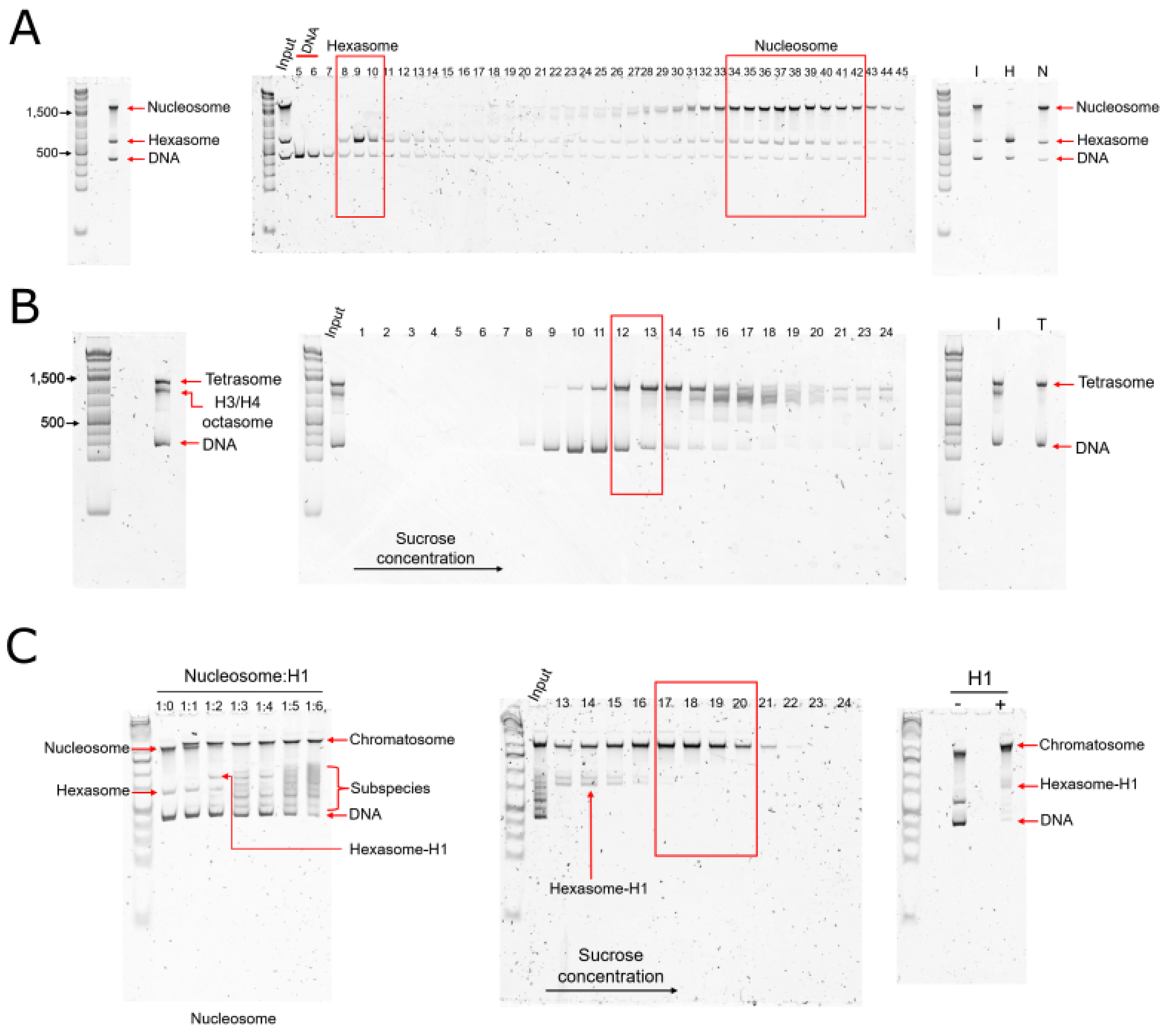
A
Input
DNA
Hexasome
Nucleosome
5 6 7 8 9 10 11 12 13 14 15 16 17 18 19 20 21 22 23 24 25 26 27 28 29 30 31 32 33 34 35 36 37 38 39 40 41 42 43 44 45
I H N
1,500
500
Nucleosome
Hexasome
DNA
B
Input
1 2 3 4 5 6 7 8 9 10 11 12 13 14 15 16 17 18 19 20 21 23 24
I T
1,500
500
Tetrasome
H3/H4 octasome
DNA
Sucrose concentration
C
Nucleosome:H1
1:0 1:1 1:2 1:3 1:4 1:5 1:6
Nucleosome
Hexasome
Chromatosome
Subspecies
DNA
Hexasome-H1
Input
13 14 15 16 17 18 19 20 21 22 23 24
Sucrose concentration
H1
- +
Chromatosome
Hexasome-H1
DNA

Fig. SI 1

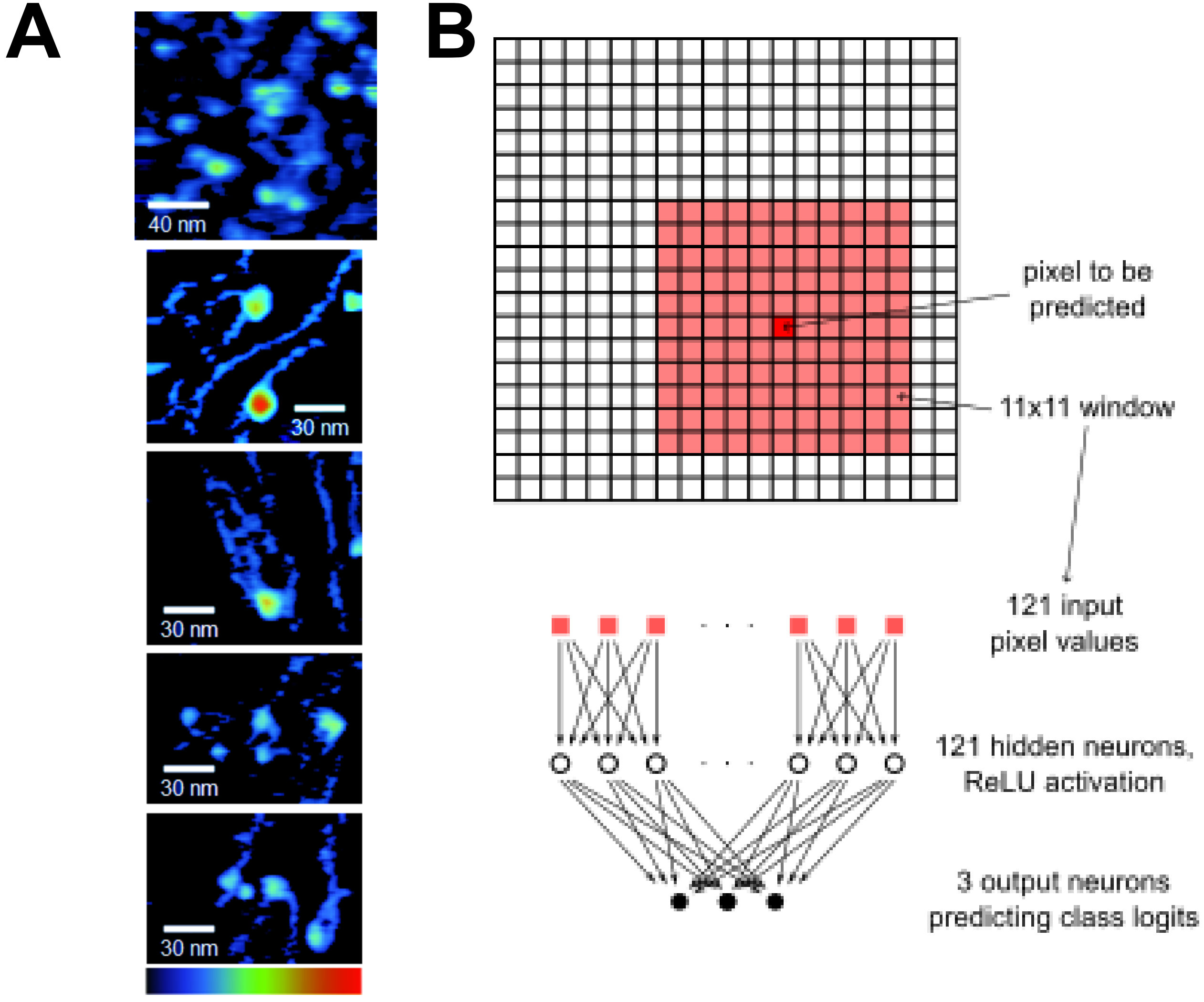
A
40 nm
30 nm
30 nm
30 nm
30 nm
B
pixel to be predicted
11x11 window
121 input pixel values
121 hidden neurons, ReLU activation
3 output neurons predicting class logits

Fig. SI 2

A

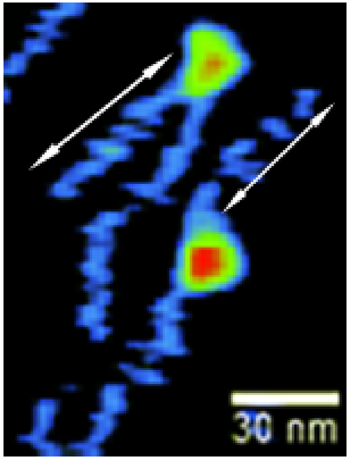
30 nm

B

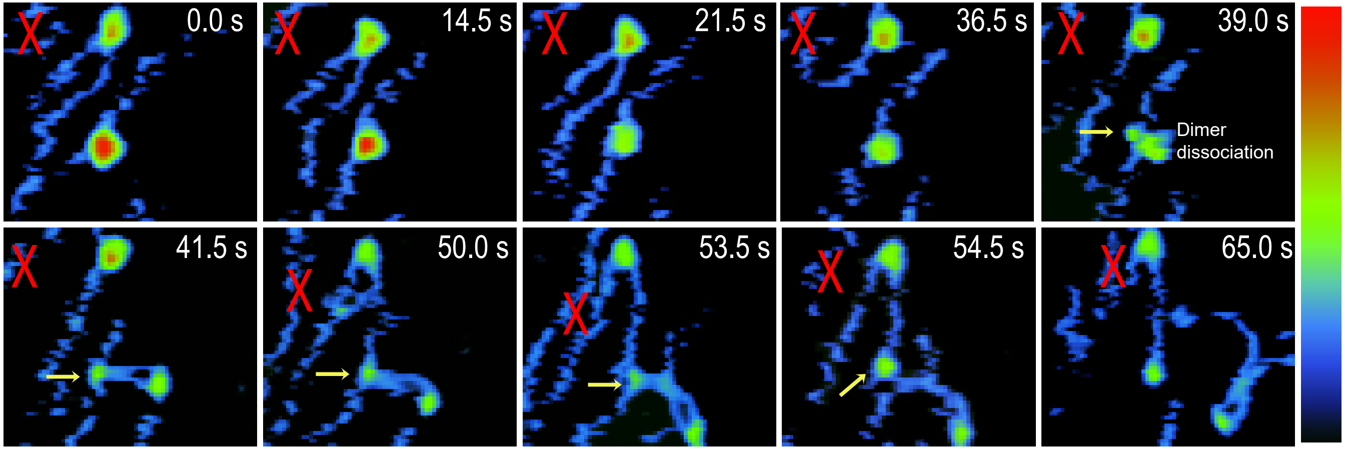
0.0 s
14.5 s
21.5 s
36.5 s
39.0 s
Dimer
dissociation
41.5 s
50.0 s
53.5 s
54.5 s
65.0 s

Fig. SI 3

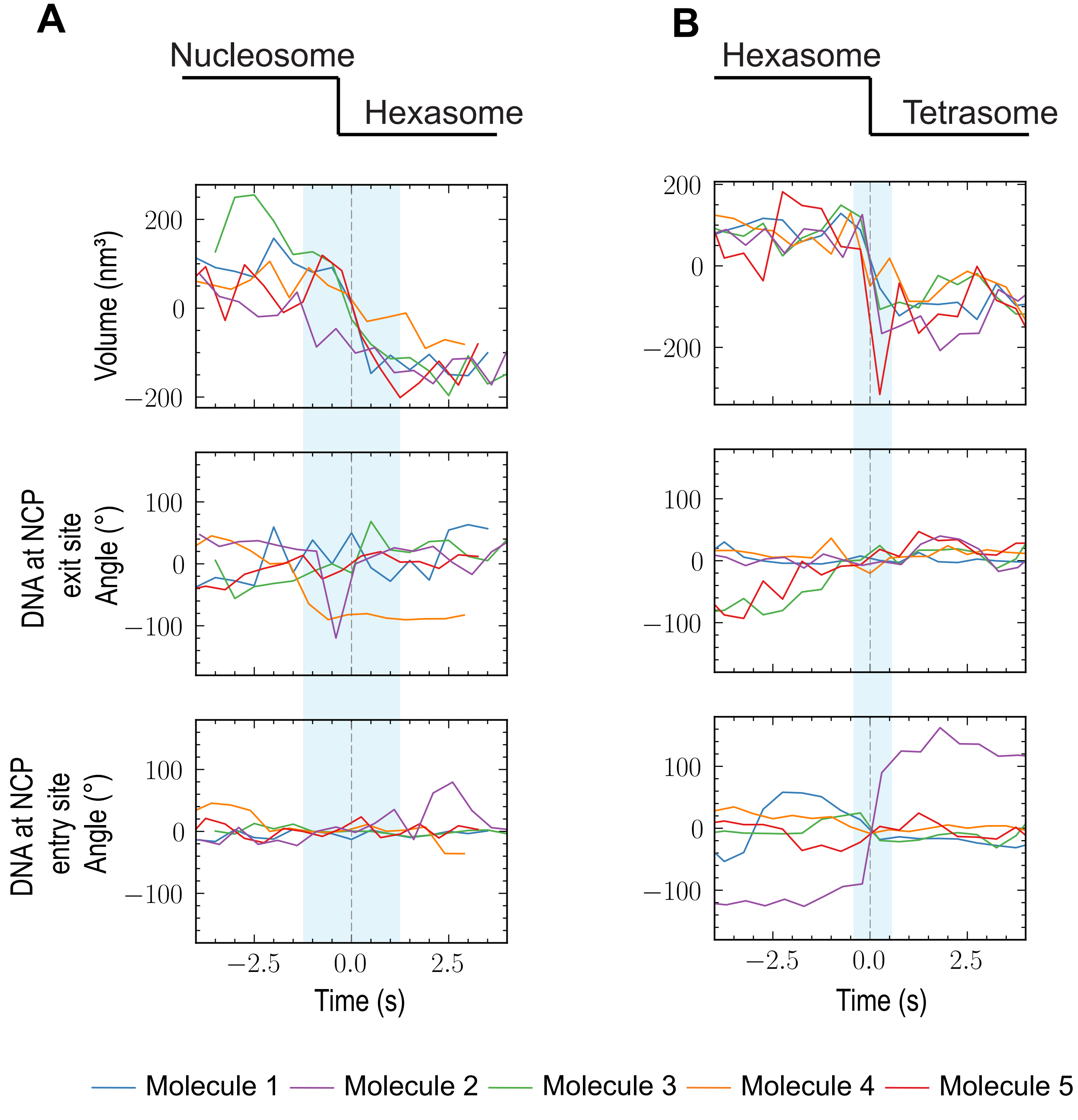

A
Nucleosome
Hexasome
B
Hexasome
Tetrasome
Volume (nm³)
DNA at NCP exit site Angle (°)
DNA at NCP entry site Angle (°)
200
0
−200
100
0
−100
−2.5
0.0
2.5
Time (s)
Molecule 1
Molecule 2
Molecule 3
Molecule 4
Molecule 5

Fig. SI 4

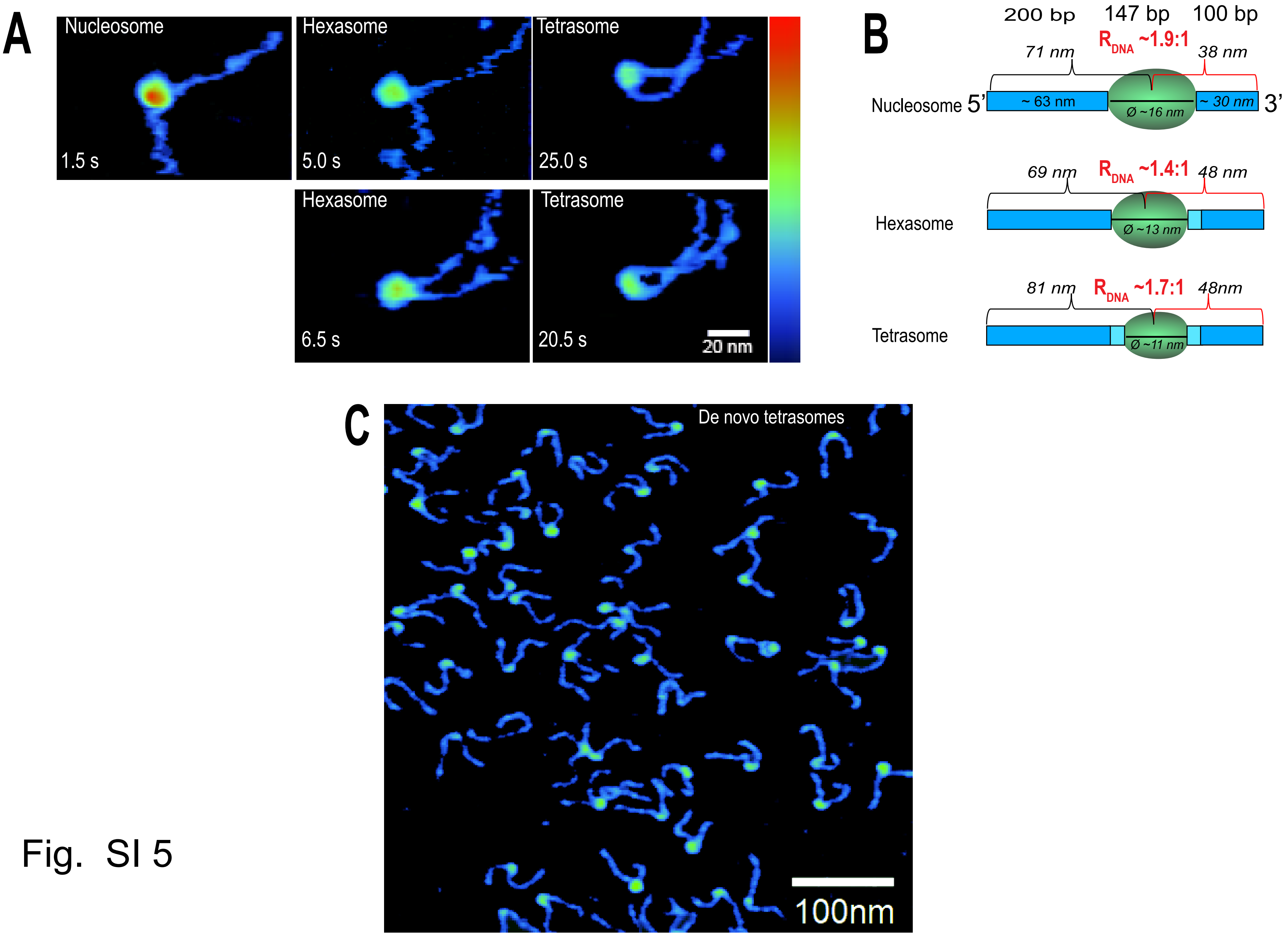
A
Nucleosome
1.5 s
Hexasome
5.0 s
Tetrasome
25.0 s
Hexasome
6.5 s
Tetrasome
20.5 s
20 nm
B
200 bp
147 bp
100 bp
R DNA ~1.9:1
71 nm
38 nm
Nucleosome 5'
~ 63 nm
Ø ~16 nm
~ 30 nm
3'
69 nm
R DNA ~1.4:1
48 nm
Hexasome
Ø ~13 nm
81 nm
R DNA ~1.7:1
48nm
Tetrasome
Ø ~11 nm
C
De novo tetrasomes
100nm

Fig. SI 5

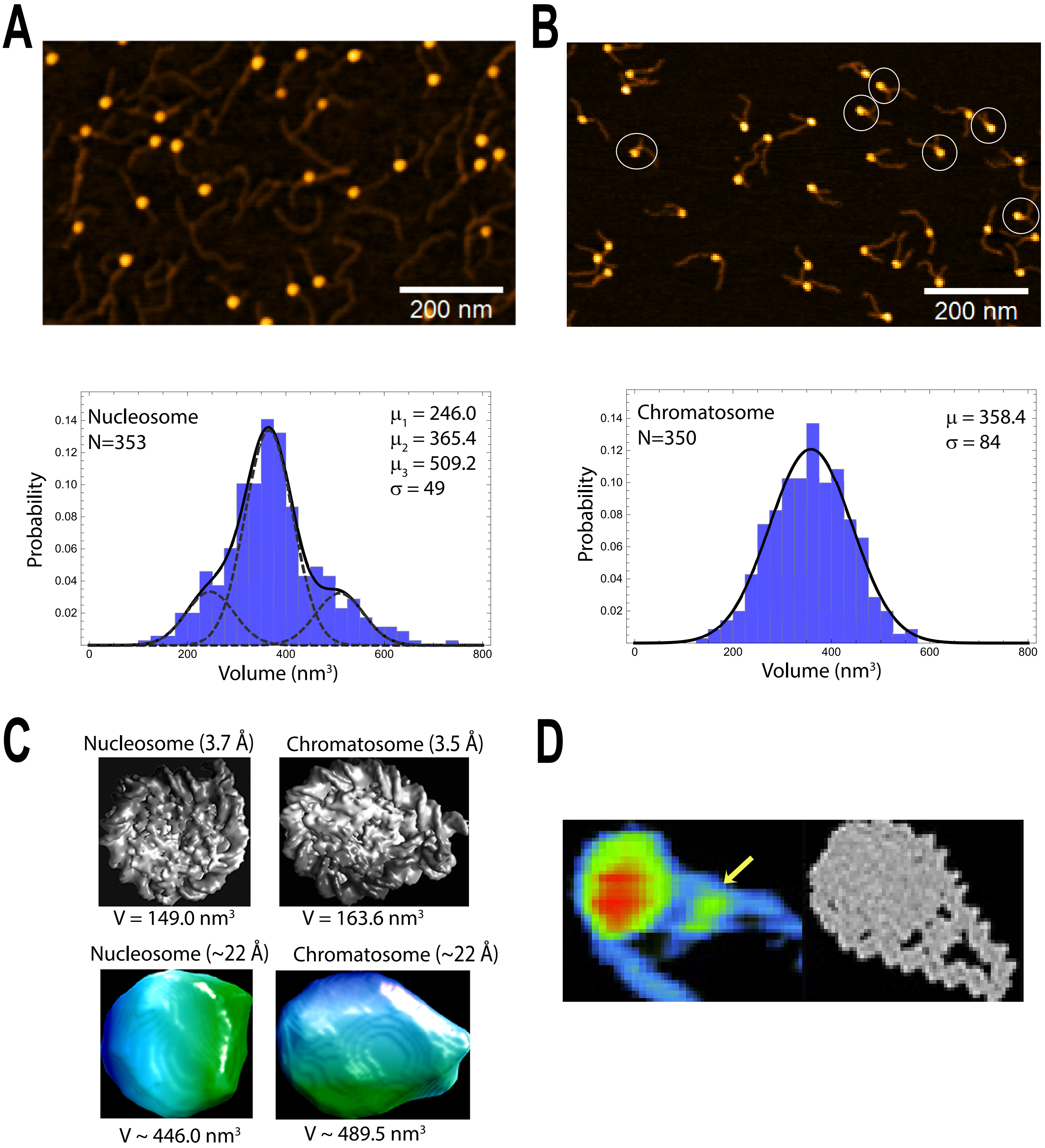
A
200 nm
B
200 nm
Nucleosome
N=353
μ1 = 246.0
μ2 = 365.4
μ3 = 509.2
σ = 49
Probability
Volume (nm3)
Chromatosome
N=350
μ = 358.4
σ = 84
C
Nucleosome (3.7 Å)
Chromatosome (3.5 Å)
V = 149.0 nm3
V = 163.6 nm3
Nucleosome (~22 Å)
Chromatosome (~22 Å)
V ~ 446.0 nm3
V ~ 489.5 nm3
D

Fig. SI 6

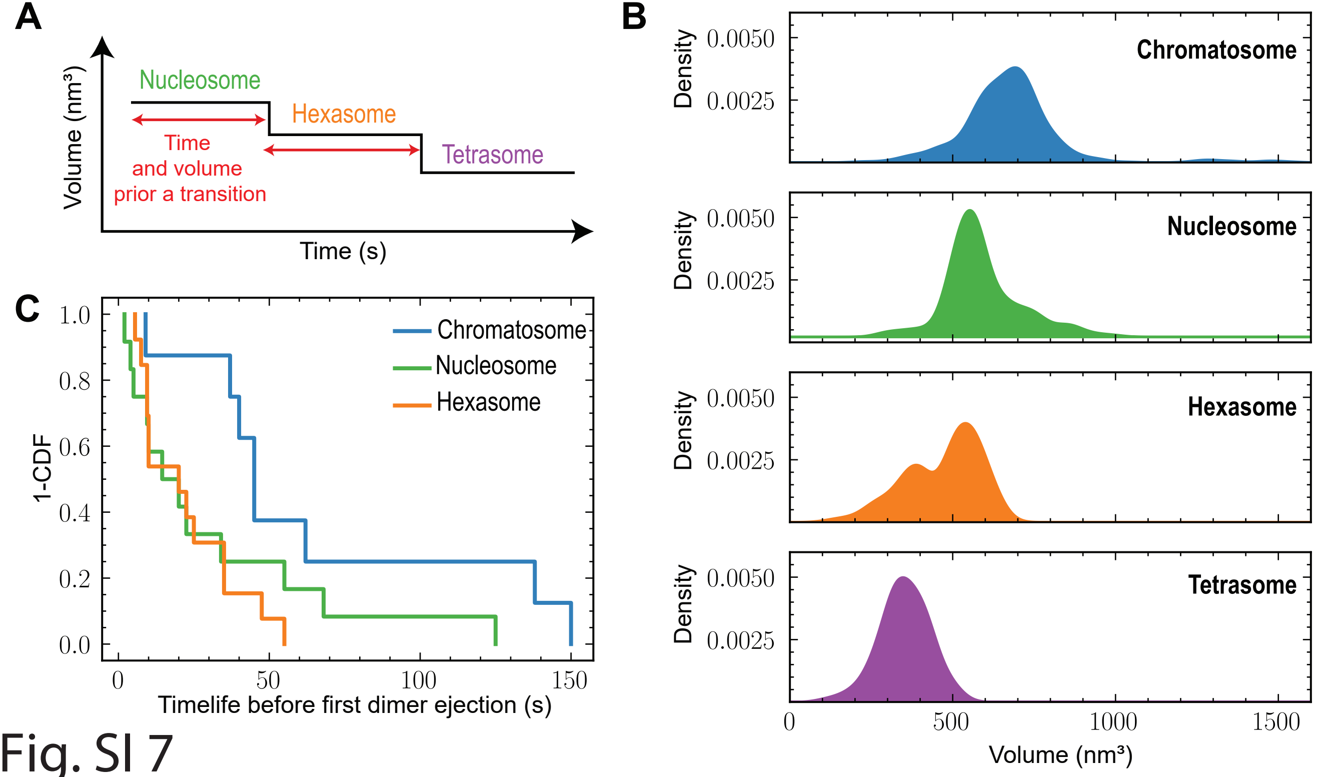
A
Volume (nm³)
Nucleosome
Hexasome
Tetrasome
Time
and volume
prior a transition
Time (s)
B
Density
0.0050
0.0025
Chromatosome
Nucleosome
Hexasome
Tetrasome
0
500
1000
1500
Volume (nm³)
C
1-CDF
1.0
0.8
0.6
0.4
0.2
0.0
Chromatosome
Nucleosome
Hexasome
0
50
100
150
Timelife before first dimer ejection (s)

Fig. SI 7

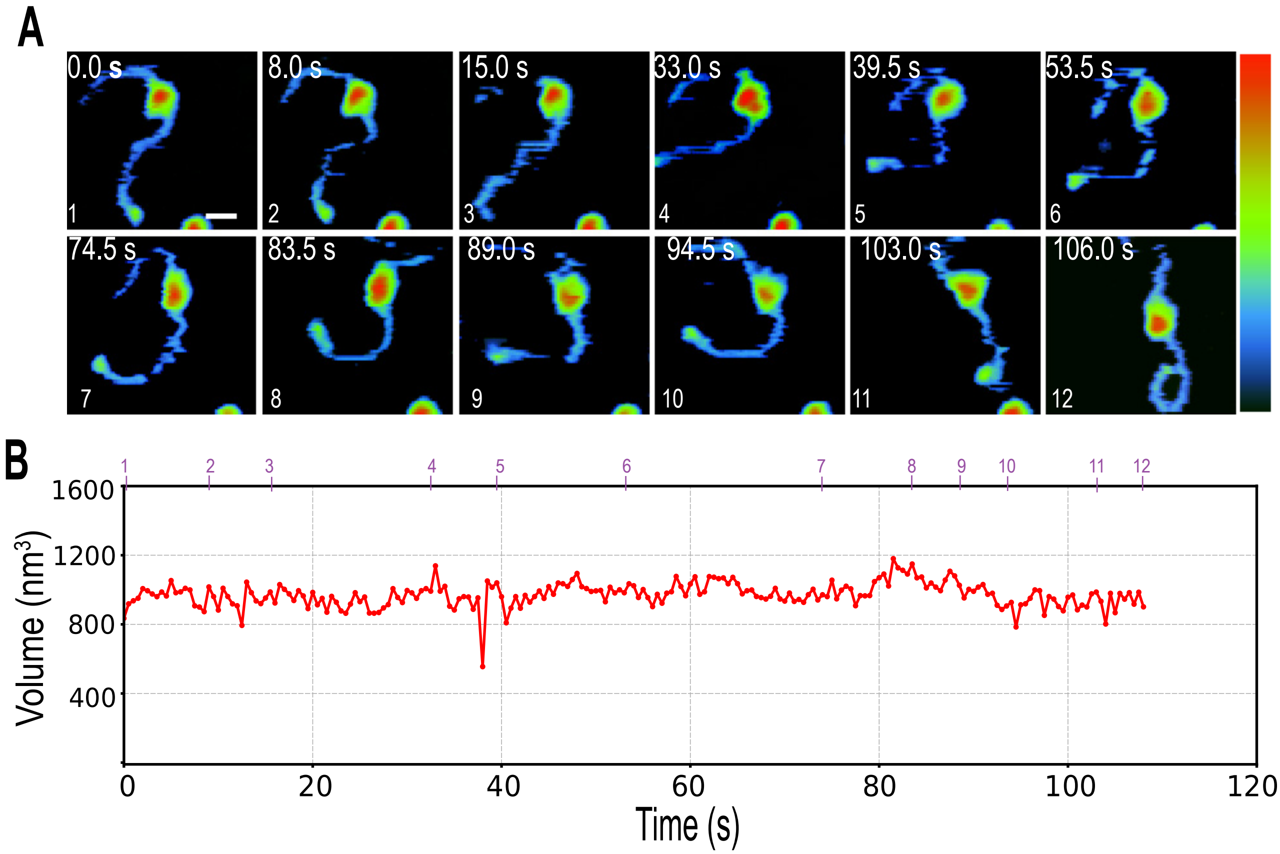
A
0.0 s
8.0 s
15.0 s
33.0 s
39.5 s
53.5 s
1
2
3
4
5
6
74.5 s
83.5 s
89.0 s
94.5 s
103.0 s
106.0 s
7
8
9
10
11
12
B
Volume (nm³)
1600
1200
800
400
0
20
40
60
80
100
120
Time (s)

Fig. SI 8

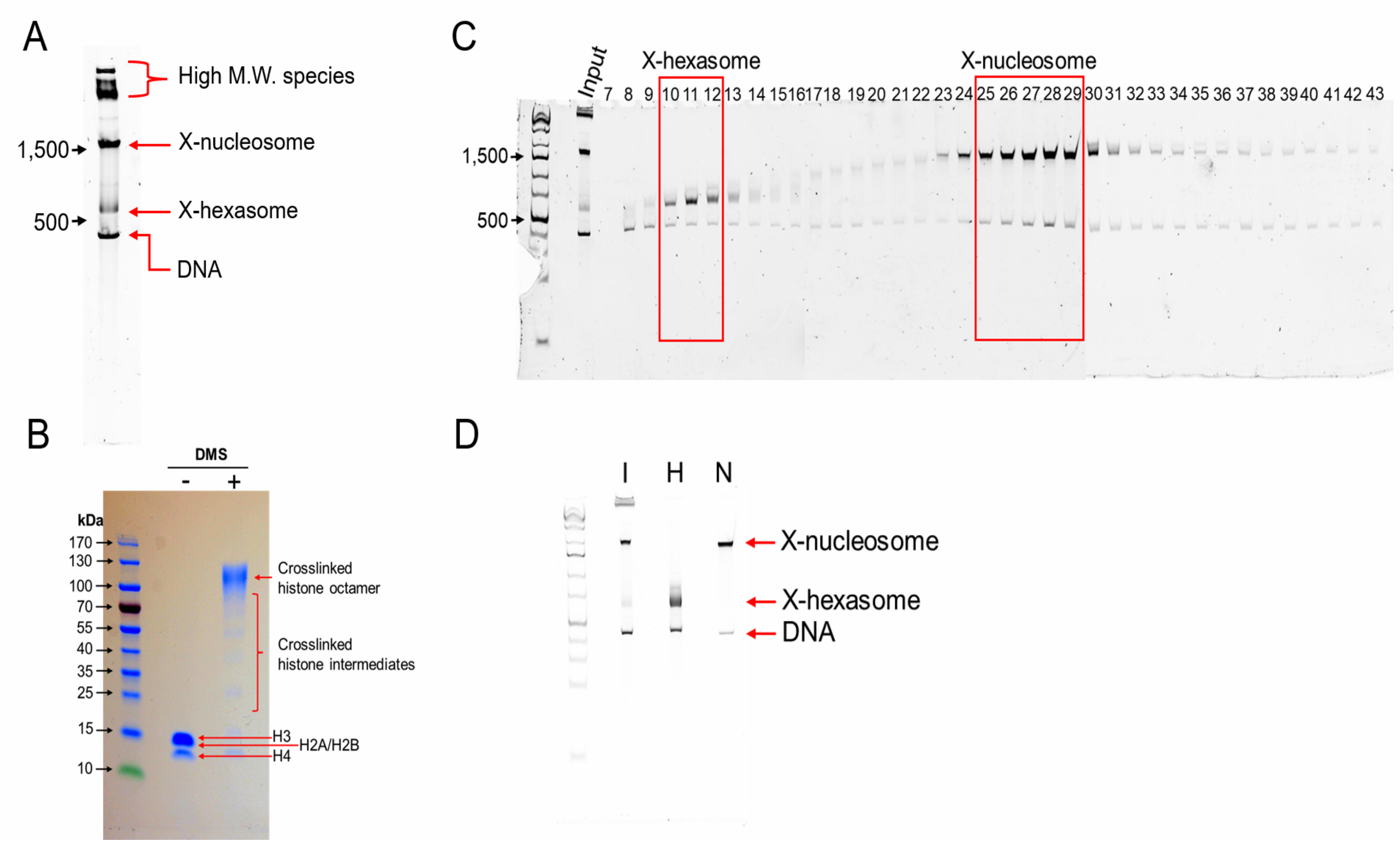
A
High M.W. species
1,500
X-nucleosome
500
X-hexasome
DNA
C
Input
X-hexasome
X-nucleosome
7 8 9 10 11 12 13 14 15 16 17 18 19 20 21 22 23 24 25 26 27 28 29 30 31 32 33 34 35 36 37 38 39 40 41 42 43
1,500
500
B
DMS
- +
kDa
170
130
100
70
55
40
35
25
15
10
Crosslinked histone octamer
Crosslinked histone intermediates
H3
H2A/H2B
H4
D
I H N
X-nucleosome
X-hexasome
DNA

Fig. SI 9

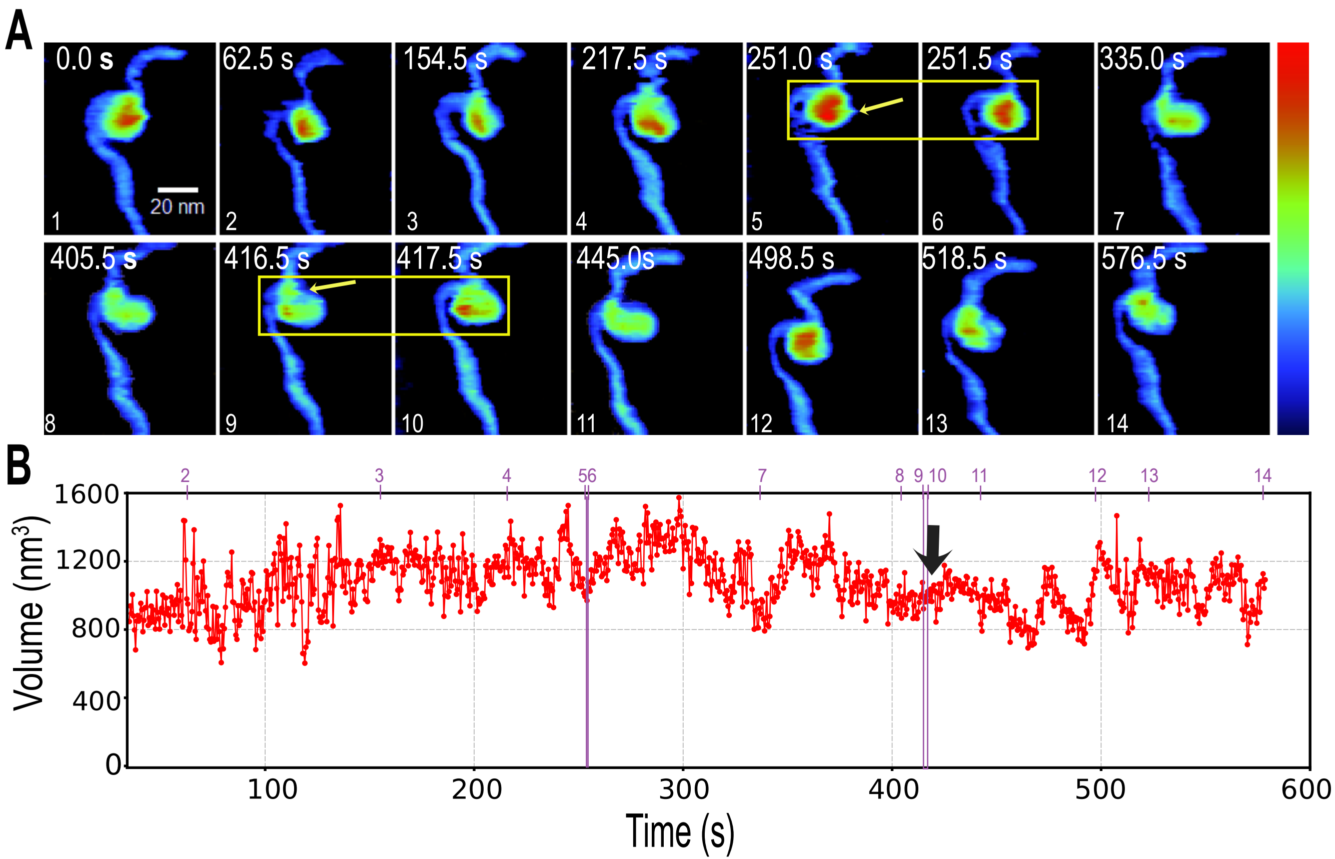
A
0.0 s
62.5 s
154.5 s
217.5 s
251.0 s
251.5 s
335.0 s
20 nm
1
2
3
4
5
6
7
405.5 s
416.5 s
417.5 s
445.0s
498.5 s
518.5 s
576.5 s
8
9
10
11
12
13
14
B
2
3
4
56
7
8
9
10
11
12
13
14
Volume (nm3)
1600
1200
800
400
0
100
200
300
400
500
600
Time (s)

Fig. SI 10

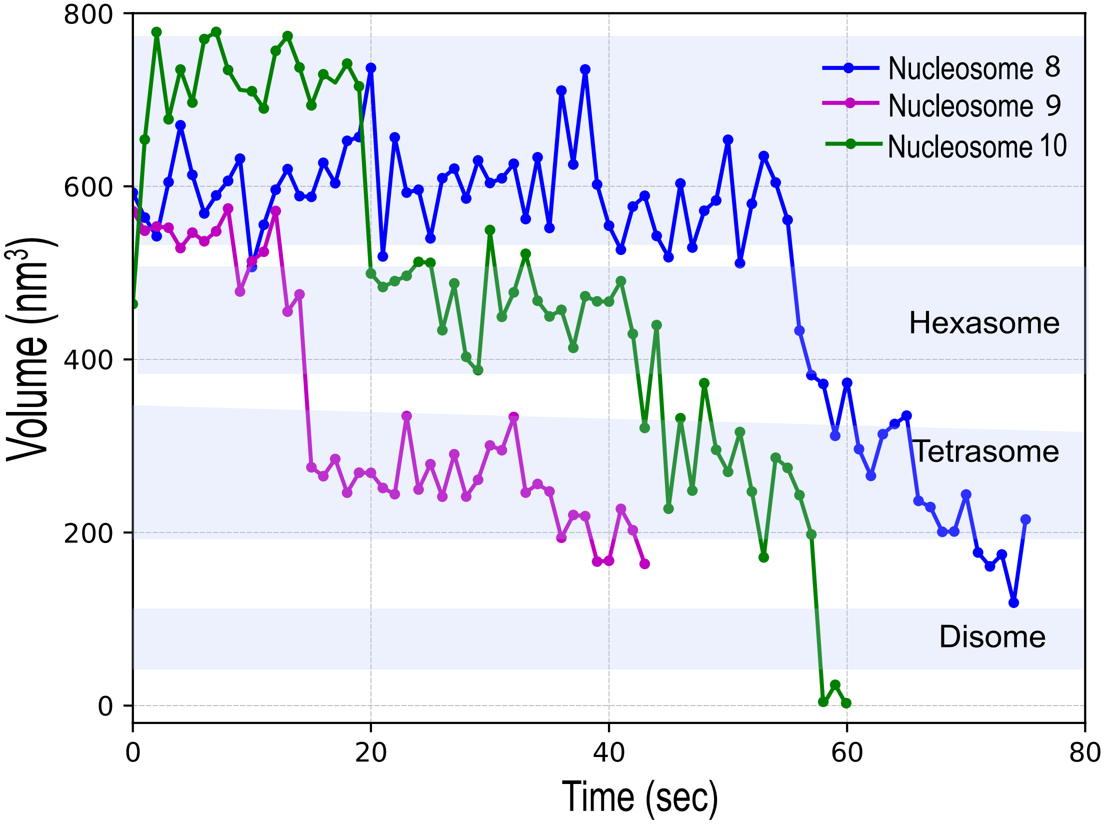

800
600
400
200
0
0
20
40
60
80
Volume (nm^3)
Time (sec)
Nucleosome 8
Nucleosome 9
Nucleosome 10
Hexasome
Tetrasome
Disome

Fig. SI 11

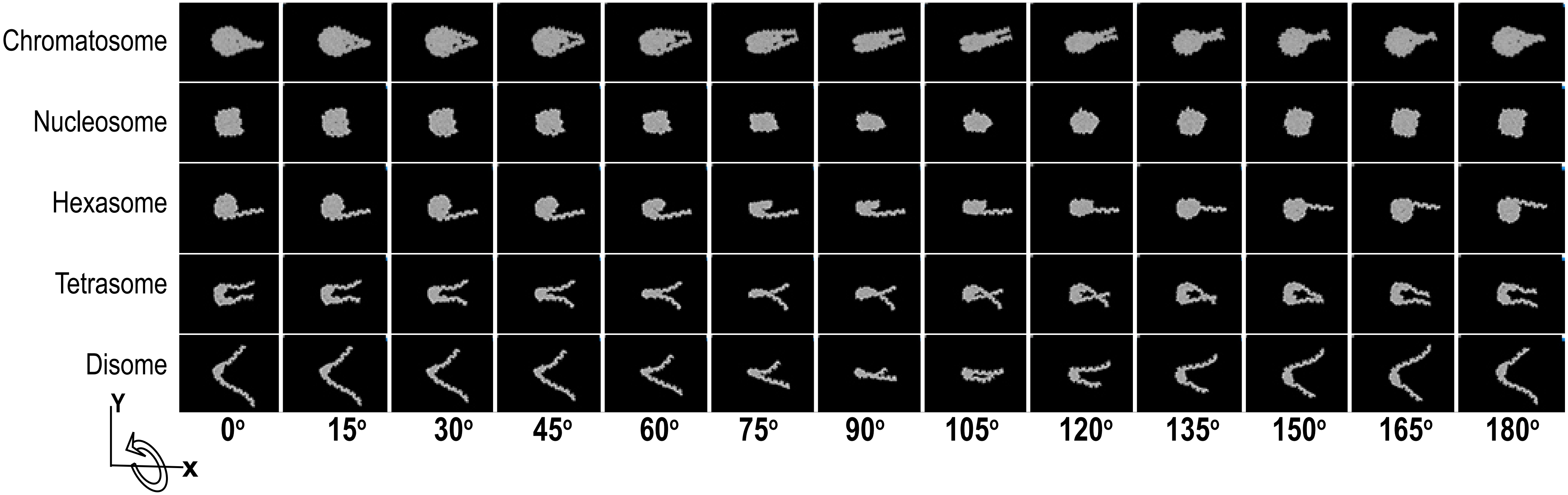
Chromatosome
Nucleosome
Hexasome
Tetrasome
Disome
Y
X
0º
15º
30º
45º
60º
75º
90º
105º
120º
135º
150º
165º
180º

Fig. SI 12
