# Supplementary material for: Real-Time Multistep Asymmetrical Disassembly of Nucleosomes and Chromatosomes Visualized by High-Speed Atomic Force Microscopy
Source: ACS Cent Sci. 2023 Dec 22;10(1):122–37. doi: 10.1021/acscentsci.3c00735 (PMC10823521; doi:10.1021/acscentsci.3c00735)
Supplement: Supplementary file 11 — oc3c00735_si_015.pdf [file oc3c00735_si_015.pdf]

oc-2023-00735u.R1

Name: Peer Review Information for "Real-time multistep asymmetrical disassembly of nucleosomes and chromatosomes visualized by High-Speed Atomic Force Microscopy"

First Round of Reviewer Comments

Reviewer: 1

Comments to the Author

In this paper, Onoa et al. provide an impressive single-molecule view on nucleosome dynamics and nucleosome disassembly. They employ an optimized HS-AFM protocol to image nucleosomes on untreated mica surfaces under near-native salt conditions in the presence of multivalent amines. Under these conditions, nucleosomes disassemble rapidly and in a stepwise fashion. Using a ML-algorithm, the authors segment the AFM images, characterizing DNA length, angles and nucleosome volume. The latter parameter, in particular, reveals stepwise decreasing volume, corresponding to dissociation of H2A-H2B dimers, formation of hexasomes, tetrasomes, disomes and finally free DNA. Intriguingly, tetrasomes are more stable than nucleosomes, and diffuse along the DNA e.g. probing the DNA for maximal stability of nucleosome formation. Importantly, the authors always compare disassembly intermediates with de-novo assembled particles, providing key controls. The authors then analyze chromatosomes, nucleosomes in complex with H1. Here, H1 stabilizes nucleosome structure by prenventing transient unwrapping and thereby modifies the nucleosome disassembly landscape.

Overall, this is a technically impressive study, giving detailed insight into nucleosome and chromatosome disassembly and of interest for the community. However, there is a large focus on the analysis of two single-molecule traces (Fig. 2B and Fig. 4B). It is not clear to me, how representative these traces and the underlying molecular behavior are. Overall, the study would be much stronger with appropriate statistics, in particular as the authors have specifically developed ML algorithms for analysis.

1. The authors analyze lifetimes of nucleosomes and hexasomes using survival distributions (~ 25 s lifetime) with ~ 10 particles each. Could this analysis be extended to other parameters? The authors state: 'Nucleosomes fully disassemble in about 50 s" or "After a brief period (typically less than 2 seconds), the heterodimer fully dissociates", or "Occasionally, the dimer dissociates". It would benefit the paper if this is more quantified (how long in average, how often % etc.). This information would then also allow comparisons with other conditions, i.e the chromatosome.

2. The authors generate a variety of nucleosomes, chromatosomes and PANS, some of them purified via preparative gel electrophoresis. However, they do not show any analytical data. This is however

important for the reader to judge the quality of the assemblies, and for serving as a benchmark for reproduction. I think the data (e.g. native gels etc.) should be included.

3. The authors claim that tetrasomes can diffuse freely on the DNA. As far as I can see, this is inferred from equal DNA length after diassembly of asymmetric nucleosomes into tetrasomes (this is however based on 2 particles) or an analysis of DNA length distributions of preassembled tetrasomes exhibiting a broad distribution. I don't think that this thus shows 'free diffusion'. This would require to see significant redistribution (e.g. using HF-AFM) of a tetramer along the DNA over many particles. What is shown is that tetrasomes can bind DNA at many positions, and might be able to redistribute but on an unknown timescale.

4. The authors state that H1 results in a smaller conformational variability. I agree that the nucleosome volume distribution has a more complex shape (trimodal) but the chromatosomes show an overall broader distibution. Thus, overall the nucleosomes might actually show less conformational variability (3 states) vs a more heterogeneous ensemble for chromatosomes.

5. The analysis of the chromatosome would again benefit from added statistics. If I am not mistaken, the authors discuss at great length one trajectory from a single molecule, but is this representative (given that the behavior is highly varied, e.g. in 4C). I realize that these are very complex experiments, but then conclusions have to be appropriately put into context.

Reviewer: 2

Comments to the Author

In this work, the authors use high-speed AFM to characterize the disassembly process of nucleosomes and chromatosomes (H1-bound nucleosomes) in real time. By measuring the changes in volume and in the orientation of the DNA strands that emerge from the nucleosome during the process, they found that disassembly occurs in a step-wise fashion that they ascribe to the sequential loss of specific histone pairs, which agrees with some models derived from other, more indirect, approaches. They validate this interpretation by examining reconstituted versions of specific incomplete nucleosomal complexes, which was indeed quite useful. In the end, they determined the

lifetime of the intermediates during the disassembly process and characterized how the process of disassembly is different in the presence of H1, which has never been done before. They also describe evidence of sliding of the tetrasomal sub-complex along the DNA (even with a positioning sequence), which also appears novel. Overall, I find that this is a very good piece of work, both in the quality of the data presented as well as (if not especially) in the analysis of the data. There are nonetheless a few concerns that I believe the authors should address.

1. The authors indicate that the lifetime of the complete nucleosomal complex before transitioning to the first intermediate is ~25 s, with the disassembly of the entire complex (including all sub-complexes) complete within a minute or two. In Figure SI 1c, they appear to indicate that the total number of nucleosomal complexes that were observed to disassemble is 9 (and that of a specific sub-complex is 13, which includes 4 from the reconstituted samples of just this sub-complex). However, there is no mention of how many complexes that they imaged that did not disassemble. I believe that this is essential information for this work. If indeed they only imaged 9 nucleosomal complexes and all 9 disassembled within two minutes of imaging, then I strongly suspect that there is an influence of the imaging tip-forces or surface adsorption on the observed process based on the simple fact that, if all of the nucleosomes disassembled within two minutes in the bulk, the authors could not possibly have prepared the sample that they imaged. That is, all of the nucleosomes would have already disassembled before imaging even began. In the least, I suspect that the time between the final dialysis of the reconstituted nucleosomes to the application to mica in the AFM is at least several minutes. High salt (> 150-200 mM) is known to induce disassembly, but the authors dialyze to ~100 mM salt (together with polyamines which stabilize the nucleosome) and then

image in a 10x dilution of this buffer. So, if anything, the buffer conditions that these authors used should have ensured a very low fraction of disassembling nucleosomes – not all of them. By contrast, if the authors, say, imaged 500 nucleosomes for 1-2 minutes each, and observed that only 9 disassembled, then a reasonable case could be made that such a low fraction in the bulk might not be generally detected. Though even with this latter case, it is important to know the length of time between the preparation of the material and the imaging of each disassembling nucleosome. That is, it would be a little suspicious if each of these 9 nucleosomes began to disassemble within 25 seconds of initially being imaged regardless of how long they were on the surface or the length of time from reconstitution or dialysis. Though if there is indeed a tip-induced effect, the process of disassembly described in this work might still be physiologically valid, just catalyzed by compressive tip forces. I have similar concerns about the observations of the disassembly of the chromatosomes as well.

2. In the discussion in the Supporting Information where the authors argue for a negligible effect of tip-forces/surface adsorption, they mention many results from other publications where tests were performed to demonstrate a negligible effect of the tip/adsorption. Did the authors perform any of the tests described in these other publications? Just because these observations were true for these other authors does not mean that they are true in the present case. The authors here do mention that they imaged at 2-fold slower rates and showed data in Fig SI 4 that reveal similar-looking trajectories for 3 nucleosomal complexes. Do the authors have statistics to bolster this claim? That is, a curve similar to Fig S1c but obtained at 2-fold slower rates. At first brush, many of the tests performed in these other publications seem more convincing than this 2-fold slower imaging of 3 complexes.

3. In Fig 1biii, the authors correctly depict that the two DNA arms emerge on the "H3/H4" side of the nucleosome. However, in Fig 1D, they show that the DNA arms emerge in the hexasome and tetrasome from the "H2/H3" side of the nucleosome. In all of these situations, both DNA arms are to the right of the core complex. It is not clear how this can occur. From movie S2, the core complex does not appear to detach and then rotate by 180°, which I believe would be necessary for the arms to maintain their right-side disposition relative to the complex and for the H3/H4 complex to go from the same side as that where the arms emerge to the opposite side. Also, in the top schematic in Fig 1D (that is, for the nucleosome), the short arm emerges from the "bottom" of the core and the long arm emerges at the "top" of the core, with each strand then continuing along in a downward and upward direction, respectively. But in their depiction of the hexasome and tetrasome, the short arm now emerges from the top of the core, and yet this arm continues downward. It is not clear, from the schematic depictions, why the short arm would not now continue upwards. Both of these comments relate to the question of whether (or how) the depicted model is physically possible. The authors may wish to add a few additional words of description.

4. The authors may wish to change the title from "spectroscopy" to "microscopy". Indeed, it seems as though the only place in the manuscript where this technique was referred to as "spectroscopy" and not "microscopy" was in the title.

Reviewer: 3

Comments to the Author

In the manuscript by Onoa and colleagues the authors use high-speed atomic force microscopy (AFM) to capture the dynamics of stepwise disassembly of nucleosomes and chromatosomes.

By using HS-AFM imaging in liquids, the authors tracked the volume of the nucleosome core particle (NCP) as a readout for disassembly events. This parameter allowed the authors to assess and characterize the AFM time lapse images into sets of disassembly events from nucleosomes to hexasomes, tetrasomes, disomes, and finally to naked DNA. By also tracking the angular changes in DNA arms, the authors described the asymmetry in the disassembly of H2A-H2B dimers with the distal dimer more poised for ejection compared to the proximal dimer. The authors imaged purified hexasomes and tetrasomes to confirm their assignment of subnucleosomal structures and disassembly dynamics. Next, the authors focused on the tetrasome to determine its position of binding and movement during disassembly. Using AFM imaging in liquid and air, they measured the lengths of DNA arms and their ratio to conclude that H3-H4 dimer diffuses freely along the DNA during disassembly. Finally, the authors imaged the spontaneous disassembly of chromatosomes and described dynamics similar to that of nucleosomes. The addition of histone H1 nonetheless seemed to provide additional stability to the core and also decreased the mobility of the DNA arms as expected.

Most of the experiments shown in the current work are well designed and provide conclusive evidence that corroborates with previous studies. As pointed out by the authors, multiple past studies have investigated the mechanism of disassembly of nucleosomes using a variety of different methods and including (HS-)AFM, sm-FRET, TR-SAXS, and molecular dynamics simulations. Indeed, the process has been described already in much detail. The main advancement of the new work lies in the imaging and the resolution of the structural details of the stepwise disassembly events as well as in the analysis of H1 containing chromatosomes.

However, some bias introduced by the use of the Widom 601 sequence and high variability between samples raise major concerns that should be addressed before being recommended for publication.

Major concerns:

1) There seems to be a high degree of variability in the duration of disassembly in the populations of both nucleosomes and chromatosomes. While the authors acknowledge this effect, it remains unclear whether this is an issue of the studied system. Other studies have described quick disassembly of nucleosomes in highly diluted conditions. How does the current work on the time scales and heterogeneity of the observation compare to other findings? The authors should address why such variability is observed.

Along this line, the degree of variability is evident, for example, in Figs. 1C and 4C. In this context, how relevant is it to interpret volume and D angle measurements from just singular samples (Fig. 1B or Fig 2B, 2D etc.)? These measurements should be displayed as an average of at least two technical replicates.

2) While the "601" positioning sequence is commonly used in this and related studies, there is concern to what degree such artificial sequence with high propensity to form nucleosomes reflects “natural” behavior of the system. For example, the “601 system” has an inherent bias with a flexible left side (more stably bound to the histone core) and a rigid right side (less stable) (PMID: 25768909). Therefore, it seems that the "601" sequence may not be a good substrate to study the dynamics of disassembly. This bias could explain the eviction of the distal H2A-H2B dimer from the rigid end, but it is unclear whether this result has any physiological relevance. To confirm that DNA unwrapping starts from the rigid side, the authors should perform a control experiment using an inverted 601 sequence by changing the rigid and flexible sides, or another experiment to remove the bias introduced by the Widom 601 sequence. Further, attempts should be undertaken to expand the findings beyond the “601 system” (i.e. by studying nucleosomes assembled on other sequences).

As for adding long DNA sequences to the ends of the nucleosomal DNA. Can it be excluded that such design does not generate levers that affect the disassembly process? After all, such long DNA sequences with free DNA ends are not found in chromatin, where nucleosomes are connected and where accessory proteins such as link er histone provide additional packaging and stabilization of the system.

3) The authors mention that DNA length could not be accurately measured due to high mobility (Pg.5, line 10). How to then assess the accuracy of the length measurements provided?

Minor concerns:

1) Pg.6, line 57: “Tracing of the short arm (flanking the 601 NPS rigid arm) and the long arm (flanking the 601 NPS flexible arm) are displayed as orange and green lines, respectively.” Isn’t the short arm green and long arm orange?

2) In Fig. 1B bottom panel, the angle of exit (theta 1) seems very fluctuating. There is a large change in angle at 10s time point which is not accompanied by any disassembly event. How relevant is it then to correlate disassembly events with angular changes?

Reviewer: 4

Comments to the Author

In this manuscript, the authors reported the real-time visualization of multistep asymmetrical disassembly of nucleosomes and chromosomes using High-Speed Atomic Force Spectroscopy. More specifically the authors have trained a neural network and developed an automatic algorithm that can track the structural changes in real time. This research could be valuable in understanding the nucleosome disassembly thereby studying the eukaryotic genome regulation. The manuscript is well written and would be interesting to the DNA and Chromosomal community. However, there are some concerns/comments the authors need to address/revise the manuscript can be accepted after incorporating them.

1. The authors used neural networks to delineate the various structural changes using AFM but the data is not provided regarding the output. The authors have mentioned ~95% prediction accuracy by training but there is no True Positive / False Positive and so on. The authors should provide a more detailed analysis of their neural network model i.e. Confusion matrix. Detailed information about machine learning is also missing. Is it supervised/unsupervised? I encourage the authors to brief about their prediction model in the main text as it is key in the prediction of dissembled structures.

2. The authors should also consider citing the important article from Kurumizaka-group http://dx.doi.org/10.1016/j.ymeth.2014.08.019 where they evaluated the nucleosome stability using thermal stability assay. The authors should also correlate their AFM observations with earlier published results briefly in the manuscript.

3. I am also curious about the intermediate structures the authors mentioned, Can the authors explain in brief about the possibilities?

Author's Response to Peer Review Comments:

Point-by-point response to the reviewers' critics:

Reviewer: 1

In this paper, Onoa et al. provide an impressive single-molecule view on nucleosome dynamics and nucleosome disassembly. They employ an optimized HS-AFM protocol to image nucleosomes on untreated mica surfaces under near-native salt conditions in the presence of multivalent amines. Under these conditions, nucleosomes disassemble rapidly and in a stepwise fashion. Using a ML-algorithm, the authors segment the AFM images, characterizing DNA length, angles and nucleosome volume. The latter parameter, in particular, reveals stepwise decreasing volume, corresponding to dissociation of H2A-H2B dimers, formation of hexasomes, tetrasomes, disomes and finally free DNA. Intriguingly, tetrasomes are more stable than nucleosomes, and diffuse along the DNA e.g. probing the DNA for maximal stability of nucleosome formation. Importantly, the authors always compare disassembly intermediates with de-novo assembled particles, providing key controls. The authors then analyze chromatosomes, nucleosomes in complex with H1. Here, H1 stabilizes nucleosome structure by prenventing transient unwrapping and thereby modifies the nucleosome disassembly landscape.
Overall, this is a technically impressive study, giving detailed insight into nucleosome and chromatosome disassembly and of interest for the community. However, there is a large focus on the analysis of two single-molecule traces (Fig. 2B and Fig. 4B). It is not clear to me, how representative these traces and the underlying molecular behavior are. Overall, the study would be much stronger with appropriate statistics, in particular as the authors have specifically developed ML algorithms for analysis.

1. The authors analyze lifetimes of nucleosomes and hexasomes using survival distributions (~ 25 s lifetime) with ~ 10 particles each. Could this analysis be extended to other parameters? The authors state: 'Nucleosomes fully disassemble in about 50 s" or "After a brief period (typically less than 2 seconds), the heterodimer fully dissociates", or "Occasionally, the dimer dissociates". It would benefit the paper if this is more quantified (how long in average, how often % etc.). This information would then also allow comparisons with other conditions, i.e the chromatosome.

Thank you very much for this fair comment. We concur with the reviewer's perspective. As a result, we have expanded the number of molecules analyzed and provided additional information on the various parameters we measured as follows: (1) Added kernel density estimator of real-time distributions of molecular volume fluctuations for chromatosomes, nucleosomes, hexasomes, and tetrasomes (Fig. SI 7B). (2) Provided new plots tracking the angular changes of each nucleosomal DNA arm at the instant of histone dimer ejection during the transitions from nucleosomes to hexasomes and from hexasomes to tetrasomes for five additional molecules (Fig. SI 4). (3) Included cumulative distribution functions for the time elapsed before the first histone ejection, comparing chromatosomes with nucleosomes and hexasomes (Fig. SI 7C). (4) Introduced a heat map displaying histone occupancy in 601 NPS of purified tetrasomes in real-time and plotted their mean square displacement for better illustration of the real-time molecular diffusion (Fig. 3E and F).

2. The authors generate a variety of nucleosomes, chromatosomes and PANS, some of them purified via preparative gel electrophoresis. However, they do not show any analytical data. This is however important for the reader to judge the quality of the assemblies, and for serving as a benchmark for reproduction. I think the data (e.g. native gels etc.) should be included.

We apologize for the oversight in not providing all our gel electrophoresis analyses. As the reviewer correctly points out, this is crucial to validate the quality and composition of each sample that underwent AFM inspection. In this revised version, we have included a comprehensive "Characterization of the assembly and purification of nucleosomes, chromatosomes, and PANS by gel electrophoresis" section in

the supporting information (SI, Pages SI 2 and SI3). Additionally, we have introduced new figures (SI 1 and SI 9) that visually depict the progression from molecule assembly, purification, to quality control of each of our newly synthesized samples.

3. The authors claim that tetrasomes can diffuse freely on the DNA. As far as I can see, this is inferred from equal DNA length after diassembly of asymmetric nucleosomes into tetrasomes (this is however based on 2 particles) or an analysis of DNA length distributions of preassembled tetrasomes exhibiting a broad distribution. I don't think that this thus shows 'free diffusion'. This would require to see significant redistribution (e.g. using HF-AFM) of a tetramer along the DNA over many particles. What is shown is that tetrasomes can bind DNA at many positions and might be able to redistribute but on an unknown timescale.

Once again, we regret the oversight in presenting our results properly. First and foremost, we would like to clarify that we did not observe free diffusion per se. Instead, we observed a confined diffusion of the histones within the 601 NPS of the tetrasomes in real-time (HS-AFM). This correction has been made in the manuscript on page 10 (line 202). We also regret that this observation was previously overlooked as it was presented as a small inset in the former Fig. 2D. In this revised version, we have highlighted this real-time observation in Fig. 3 by adding two new panels (3E and 3F). Fig. 3E now displays selected frames where histone movement is evident, with an overlap of the aligned DNA and histone positions represented by colored circles, along with a heat map illustrating the preferred occupancy of the dimers in the DNA curvature. In Fig. 3F, the mean square displacement of the histones is presented, confirming that the diffusion is indeed confined. We have described this observation on page 10 (lines 202-209), and we transparently acknowledge that this type of observation was challenging to capture, as it required the DNA arms to separate sufficiently to allow movement, which was not the case in most of our observations. Additionally, we have included a supporting movie (Movie SI 6) that demonstrates how the DNA arms of purified tetrasomes remained in close proximity for an extended period of time. This property also imparts them with resilience to disassembly. Therefore, based on our robust data from 130 static nucleosomes where the DNA arms appear well separated at the time of binding to the surface and exhibited highly variable R_{DNA}*, along with our observation of histone movement in real-time, we conclude that the* $(H3.H4)_2$ *tetramer has the potential for diffusion within the nucleosome positioning sequence. We have also corrected our conclusion on page 19 (lines 432-435).*

4. The authors state that H1 results in a smaller conformational variability. I agree that the nucleosome volume distribution has a more complex shape (trimodal) but the chromatosomes show an overall broader distibution. Thus, overall the nucleosomes might actually show less conformational variability (3 states) vs a more heterogeneous ensemble for chromatosomes.

We appreciate the reviewer's insightful observation. Following a thorough visual examination and a comparison of the volume distributions measured for both static and dynamic chromatosomes and nucleosome histone cores, we concur with the reviewer's assessment. In both instances, we have identified that the volume distributions of chromatosomes are indeed broader than those of nucleosomes, suggesting heterogeneous ensemble of conformations in chromatosomes. Furthermore, upon meticulous scrutiny of our videos, it becomes apparent that chromatosomes exhibit lobed conformations more frequently than nucleosomes, as illustrated in Figures 1C and 4C. This observation has been incorporated into the revised manuscript on pages 14 (lines 298-300) and 15-16 (lines 352-364).

5. The analysis of the chromatosome would again benefit from added statistics. If I am not mistaken, the authors discuss at great length one trajectory from a single molecule, but is this representative (given that

the behavior is highly varied, e.g. in 4C). I realize that these are very complex experiments, but then conclusions have to be appropriately put into context.

As mentioned in our responses to Comment #1 and Comment #4, we have broadened our analysis to encompass more molecules and have introduced the new Figure SI 7. Our examination of the time elapsed before the first ejection event in chromatosomes offers additional validation of the notion that the presence of the linker histone prolongs the lifetime of nucleosomes. We have now included a new paragraph on page 16 (Lines 367-374) to articulate this result.

Reviewer: 2

In this work, the authors use high-speed AFM to characterize the disassembly process of nucleosomes and chromatosomes (H1-bound nucleosomes) in real time. By measuring the changes in volume and in the orientation of the DNA strands that emerge from the nucleosome during the process, they found that disassembly occurs in a step-wise fashion that they ascribe to the sequential loss of specific histone pairs, which agrees with some models derived from other, more indirect, approaches. They validate this interpretation by examining reconstituted versions of specific incomplete nucleosomal complexes, which was indeed quite useful. In the end, they determined the lifetime of the intermediates during the disassembly process and characterized how the process of disassembly is different in the presence of H1, which has never been done before. They also describe evidence of sliding of the tetrasomal sub-complex along the DNA (even with a positioning sequence), which also appears novel. Overall, I find that this is a very good piece of work, both in the quality of the data presented as well as (if not especially) in the analysis of the data. There are nonetheless a few concerns that I believe the authors should address.

1. The authors indicate that the lifetime of the complete nucleosomal complex before transitioning to the first intermediate is ~25 s, with the disassembly of the entire complex (including all sub-complexes) complete within a minute or two. In Figure SI 1c, they appear to indicate that the total number of nucleosomal complexes that were observed to disassemble is 9 (and that of a specific sub-complex is 13, which includes 4 from the reconstituted samples of just this sub-complex). However, there is no mention of how many complexes that they imaged that did not disassemble. I believe that this is essential information for this work. If indeed they only imaged 9 nucleosomal complexes and all 9 disassembled within two minutes of imaging, then I strongly suspect that there is an influence of the imaging tip-forces or surface adsorption on the observed process based on the simple fact that, if all of the nucleosomes disassembled within two minutes in the bulk, the authors could not possibly have prepared the sample that they imaged. That is, all of the nucleosomes would have already disassembled before imaging even began. In the least, I suspect that the time between the final dialysis of the reconstituted nucleosomes to the application to mica in the AFM is at least several minutes. High salt (> 150-200 mM) is known to induce disassembly, but the authors dialyze to ~100 mM salt (together with polyamines which stabilize the nucleosome) and then image in a 10x dilution of this buffer. So, if anything, the buffer conditions that these authors used should have ensured a very low fraction of disassembling nucleosomes – not all of them. By contrast, if the authors, say, imaged 500 nucleosomes for 1-2 minutes each, and observed that only 9 disassembled, then a reasonable case could be made that such a low fraction in the bulk might not be generally detected. Though even with this latter case, it is important to know the length of time between the preparation of the material and the imaging of each disassembling nucleosome. That is, it would be a little suspicious if each of these 9 nucleosomes began to disassemble within 25 seconds of initially being imaged regardless of how long they were on the surface or the length of time from reconstitution or dialysis. Though if there is indeed a tip-induced effect, the process of disassembly described in this work might still be physiologically valid, just

catalyzed by compressive tip forces. I have similar concerns about the observations of the disassembly of the chromatosomes as well.

We appreciate the reviewer's legitimate concerns and the meticulous analysis presented to help interpret our results within the context of tip-sample interactions affecting the stability of nucleosomal complexes. The reviewer correctly notes that the nucleosomes survive the purification and dialysis process, which involves bulk manipulations at micromolar concentrations. However, even at this relatively high concentration, our electrophoresis analysis (New Fig. SI 1) reveals that a portion of nucleosomes transitions to hexasomes and bare DNA. For our AFM experiments, we must utilize miniaturized mica substrates (1.5 mm in diameter), necessitating a sample dilution from 200 to 900-fold. It is well-documented that nucleosome stability is concentration-dependent, even within the context of nucleosome arrays (ref.10 and 12). We believe this concentration variable is crucial to consider, as our sample may have consisted of a mixture of species with metastable configurations prior to deposition and AFM scanning. We have provided an account of these significant considerations on pages 3 (lines 11 to the end) and 4 (lines 15-17). While we acknowledge the potential effects of the tip during scanning (as mentioned on page 4, lines 44-47), we were committed to addressing the complexities of our experimental setup.

Recognizing the importance of these experimental conditions and aiming for enhanced transparency in our approach, we have now incorporated additional details about the dilution conditions and acquisition practices in the high-speed AFM subsection of the Methods (Pg., 21, lines531 and 535-538). Additionally, we have introduced a new figure (SI 2A) featuring AFM images of various surfaces observed before the selection of a suitable molecule for extensive scanning. These images illustrate the coexistence of different species, including nucleosomes, PANS, and bare DNA, even after 1 or 2 hours of deposition. This suggests that, under our experimental conditions, a fraction of nucleosomes can survive intact, which implies they would not disassemble rapidly unless catalyzed, as suggested by the reviewer, and which could also be attributed to tip-induced forces. However, we propose that these tip-induced forces may primarily influence DNA mobility, facilitating DNA unwrapping, rather than directly triggering histone dislodging. If it were the latter, the predominant end product of disassembly would likely be bare DNA at our experimental time scales. Nevertheless, our results consistently indicate that the process significantly decelerates at the tetrasome state (as seen in Figs. 1C, 2A, 4A, 4C, SI 3). Both pure tetrasomes and those formed during the disassembly process can endure for minutes under the same AFM experimental conditions.

To emphasize these observations, we have incorporated additional statements in pertinent sections of the manuscript, specifically on pages 3, 4, and 17. These additions are intended to underscore our interpretation of the disassembly process and the potential influence of tip-induced forces.

2. In the discussion in the Supporting Information where the authors argue for a negligible effect of tip-forces/surface adsorption, they mention many results from other publications where tests were performed to demonstrate a negligible effect of the tip/adsorption. Did the authors perform any of the tests described in these other publications? Just because these observations were true for these other authors does not mean that they are true in the present case. The authors here do mention that they imaged at 2-fold slower rates and showed data in Fig SI 4 that reveal similar-looking trajectories for 3 nucleosomal complexes. Do the authors have statistics to bolster this claim? That is, a curve similar to Fig S1c but obtained at 2-fold slower rates. At first brush, many of the tests performed in these other publications seem more convincing than this 2-fold slower imaging of 3 complexes.

We appreciate the reviewer's valid concern, which is related to the previous point. It is important to clarify that we cannot determine the net impact of tip-force on the disassembly process, and that we are not ruling out its potential effects. Our argument is centered on the notion that the structural and specific dynamic

characteristics of the molecule are the primary drivers of the entire process. Furthermore, in addition to: (i) replicating the uni/bimodal and rapid disassembly of nucleosomes using positively charged mica and buffers containing divalent metal cations (no particularly useful for our purposes), (ii) variations in scan rate (which show no qualitatively or quantitative differences), and (iii) lifting the cantilever on and off the surface, we have demonstrated that different types of molecules, such as chromatosomes and tetrasomes, exhibit significantly different lifetimes compared to nucleosomes when scanned under similar conditions. This further emphasizes the importance of the inherent properties of the molecules in influencing the observed dynamics.

Nonetheless, we took seriously the reviewer's concerns and conducted new experiments to investigate our hypothesis, which posits that specific structural arrangements within the molecules are the primary driving force behind their disassembly. To test this, we conducted two experiments: (1) We inhibited nucleosomal DNA unwrapping and NCP reorganization using formaldehyde. (2) We partially impaired histone displacement with dimethyl suberimidate (DMS). Subsequently, we subjected these nucleosomes to extensive and rigorous AFM scanning, increasing the tip-force to the point of physically deforming the NCP.

In both scenarios, these nucleosomes exhibited a partial retention of their intrinsic dynamics, enduring scanning for up to 10 minutes without any apparent evidence of molecular dissociation. Instead, what we observed was the molecules quickly recovering from force spikes within a second. These findings are now reported in the revised manuscript on pages 16 and 17, and we have included three new figures in the Supporting Information (New Figs. SI 8, 9, and 10 and Movies SI 8 and SI 9).

As such, we reaffirm our initial observation and the conclusion stated on page 17: "Our findings, combined with those published by others, suggest that nucleosome disassembly results from the synergistic influence of the specific molecule's composition, structure, elasticity, and the AFM environment. A crucial factor may be the heterogeneity in the nucleosome's structure, induced by the substantial dilution necessary for HS-AFM, resulting in varying degrees of wrapping and subsequent histone core rearrangements, which, to a certain extent, dictate its stability on the mica."

3. In Fig 1biii, the authors correctly depict that the two DNA arms emerge on the “H3/H4” side of the nucleosome. However, in Fig 1D, they show that the DNA arms emerge in the hexasome and tetrasome from the “H2/H3” side of the nucleosome. In all of these situations, both DNA arms are to the right of the core complex. It is not clear how this can occur. From movie S2, the core complex does not appear to detach and then rotate by 180°, which I believe would be necessary for the arms to maintain their right-side disposition relative to the complex and for the H3/H4 complex to go from the same side as that where the arms emerge to the opposite side. Also, in the top schematic in Fig 1D (that is, for the nucleosome), the short arm emerges from the “bottom” of the core and the long arm emerges at the “top” of the core, with each strand then continuing along in a downward and upward direction, respectively. But in their depiction of the hexasome and tetrasome, the short arm now emerges from the top of the core, and yet this arm continues downward. It is not clear, from the schematic depictions, why the short arm would not now continue upwards. Both of these comments relate to the question of whether (or how) the depicted model is physically possible. The authors may wish to add a few additional words of description.

We apologize for any confusion that our cartoon representation of the nucleosomes captured by HS-AFM may have caused. In our depiction, we illustrated the nucleosome as a particle comprising eight components, formed by two H2A·H2B dimers and two H3·H4 dimers. Consequently, there is a H2A·H2B dimer at the top and another at the bottom of the complex. In Figure 1D, we show the DNA arms emerging from the H3·H4 dimer at the top of the hexasome, which only possesses a bottom H2A·H2B dimer. This

DNA crosses the wrapped DNA in contact with the bottom H2A·H2B dimer but is not in direct contact with it. Therefore, there is no H2/H3 exit in this context. To clarify this confusion, we have updated the cartoons in figure 1. We rotated the nucleosome in figure 1B (iii) to have the dyad at same location as that in the tetrasome in Fig. 1D. Additionally, we changed the colors of the DNA arms to match the assignment of the angles and used a dashed line to represent the arm underneath the crossing of the nucleosomal DNA. Furthermore, we have described the orientation of our cartoonish nucleosome on page 7, lines 110-111.

4. The authors may wish to change the title from "spectroscopy" to "microscopy". Indeed, it seems as though the only place in the manuscript where this technique was referred to as "spectroscopy" and not "microscopy" was in the title.

We appreciate the keen observation made by the reviewer, which eluded our attention. We have promptly updated the titles accordingly.

Reviewer: 3

In the manuscript by Onoa and colleagues the authors use high-speed atomic force microscopy (AFM) to capture the dynamics of stepwise disassembly of nucleosomes and chromatosomes.
By using HS-AFM imaging in liquids, the authors tracked the volume of the nucleosome core particle (NCP) as a readout for disassembly events. This parameter allowed the authors to assess and characterize the AFM time lapse images into sets of disassembly events from nucleosomes to hexasomes, tetrasomes, disomes, and finally to naked DNA. By also tracking the angular changes in DNA arms, the authors described the asymmetry in the disassembly of H2A-H2B dimers with the distal dimer more poised for ejection compared to the proximal dimer. The authors imaged purified hexasomes and tetrasomes to confirm their assignment of subnucleosomal structures and disassembly dynamics. Next, the authors focused on the tetrasome to determine its position of binding and movement during disassembly. Using AFM imaging in liquid and air, they measured the lengths of DNA arms and their ratio to conclude that H3-H4 dimer diffuses freely along the DNA during disassembly. Finally, the authors imaged the spontaneous disassembly of chromatosomes and described dynamics similar to that of nucleosomes. The addition of histone H1 nonetheless seemed to provide additional stability to the core and also decreased the mobility of the DNA arms as expected.
Most of the experiments shown in the current work are well designed and provide conclusive evidence that corroborates with previous studies. As pointed out by the authors, multiple past studies have investigated the mechanism of disassembly of nucleosomes using a variety of different methods and including (HS-AFM, sm-FRET, TR-SAXS, and molecular dynamics simulations). Indeed, the process has been described already in much detail. The main advancement of the new work lies in the imaging and the resolution of the structural details of the stepwise disassembly events as well as in the analysis of H1 containing chromatosomes.
However, some bias introduced by the use of the Widom 601 sequence and high variability between samples raise major concerns that should be addressed before being recommended for publication.

Major concerns:
1) There seems to be a high degree of variability in the duration of disassembly in the populations of both nucleosomes and chromatosomes. While the authors acknowledge this effect, it remains unclear whether this is an issue of the studied system. Other studies have described quick disassembly of nucleosomes in highly diluted conditions. How does the current work on the time scales and heterogeneity of the observation compare to other findings? The authors should address why such variability is observed.

Along this line, the degree of variability is evident, for example, in Figs. 1C and 4C. In this context, how relevant is it to interpret volume and D angle measurements from just singular samples (Fig. 1B or Fig 2B, 2D etc.)? These measurements should be displayed as an average of at least two technical replicates.

Thank you for the insightful comments that relate to the concerns raised by reviewer 1 who also pinpointed the challenges associated with sample purification and manipulations as well as reviewer 2, who has expressed concern about tip artifacts.

Our purified nucleosome samples typically contain enriched intact octameric structures, but they also exhibit a small, albeit slowly increasing, amount of partially assembled nucleosomes (PANS), mainly hexasomes, as evidenced in the new Fig. SI 1A. This implies that, even at concentrations 2.5 orders of magnitude higher than those necessary for HS-AFM, these nucleosomes might undergo structural rearrangements and even disassembly in solution. A large dilution of the sample further reduces their stability, making them more prone to forming metastable and partially assembled species (ref. 10 and 12). Additionally, the uncontrolled adsorption of these molecules onto a charged surface likely results in a wide range of molecular orientations, further compromising structural stability. Consequently, it is highly probable that structural heterogeneity within the molecules exists even before the imaging process begins. In our Methods section, we have elaborated on our approach, emphasizing that we confined our analysis to isolated molecules that appear to be intact nucleosomes (Pg., 21). In this revised manuscript, we have taken steps to underscore this point further by introducing a new figure (SI 2A), which showcases several surfaces from different depositions, revealing the coexistence of various species before focusing our observations on individual nucleosomes. We have also summarized this explanation on pages 3-4 and explicitly attributed the variability in the duration of the molecules on the surface to the intrinsic structural heterogeneity of the samples, emphasizing this on page 6(lines 84-86). Nonetheless, we do not disregard the potential influence of the scanning tip (Pg., 4, lines 44-47) catalyzing the disassembly process.

We address the comparison of our results with those reported by other authors on page 17 (lines 400 to 424). It is essential to highlight that there is a scarcity of statistical analysis in the literature regarding comparable real-time dynamics of nucleosomes. Consequently, making detailed comparisons with existing studies is challenging.

In response to the reviewer's valid request for validation of our interpretation regarding the unwrapping events observed during disassembly, we have introduced a new figure (SI 4) illustrating the relationship between angular changes and volume at the instant of histone ejection. The intricate dynamics of the DNA arms are intertwined with the dynamical complexity within the entire frame, including collisions with neighboring molecules, NCP displacements, sudden conformational changes in the core, and even transient interactions with the AFM tip. Consequently, accurately tracking the angular changes of the DNA throughout the entire observation is exceptionally challenging. We have now provided a comprehensive disclosure of these technical limitations in our Methods section (Pg., 23). Lastly, to ensure the accuracy of the data presented in Figure SI 4, manual corrections were applied as needed, and we have a high level of confidence in these results. We have also included an additional paragraph on page 7 (lines 119-130) to describe our new observations, which align nicely with existing data.

2) While the "601" positioning sequence is commonly used in this and related studies, there is concern to what degree such artificial sequence with high propensity to form nucleosomes reflects “natural” behavior of the system. For example, the “601 system” has an inherent bias with a flexible left side (more stably bound to the histone core) and a rigid right side (less stable) (PMID: 25768909). Therefore, it seems that the "601" sequence may not be a good substrate to study the dynamics of disassembly. This bias could explain the eviction of the distal H2A-H2B dimer from the rigid end, but it is unclear whether this result has any physiological relevance. To confirm that DNA unwrapping starts from the rigid side, the authors should perform a control experiment using an inverted 601 sequence by changing the rigid and flexible sides, or another experiment to remove the bias introduced by the Widom 601 sequence. Further, attempts

should be undertaken to expand the findings beyond the “601 system” (i.e. by studying nucleosomes assembled on other sequences).
As for adding long DNA sequences to the ends of the nucleosomal DNA. Can it be excluded that such design does not generate levers that affect the disassembly process? After all, such long DNA sequences with free DNA ends are not found in chromatin, where nucleosomes are connected and where accessory proteins such as linker histone provide additional packaging and stabilization of the system.

While we acknowledge the reviewer's point about the artificial nature of the 601 NPS, we respectfully hold a different perspective regarding the physiological relevance of the results obtained using this system. The 601 NPS, since its discovery, has been widely employed for in vitro studies of nucleosomes. These studies have yielded crucial insights into the structural, biochemical, and mechanistic aspects of nucleosomes and their interactions with enzymes. Additionally, it's noteworthy that recently available structures of nucleosomes assembled on native sequences demonstrate that the nucleosome architecture remains relatively consistent with what was initially reported for the Widom 601 sequence (ref. 43 and 44). However, it's reasonable to expect that the energetics associated with processes like DNA unwrapping may differ and depend on the DNA sequence, as indicated by Dnase I sensitivity assays (ref.43). The DNA sequence's influence on mechanical flexibility can also extend to the propensity of one DNA arm to unwrap before the other, as demonstrated by several laboratories. Importantly, this asymmetry in DNA flexibility is not exclusive to artificial sequences but also exists in native sequences.

That being said, we believe that the fundamental contributions and methodologies developed in this study have the potential to be extended to other nucleosomes assembled on native sequences, as well as to explore various histone variants and epigenetic modifications in future research endeavors beyond the scope of this study. To address the reviewer's comment, we have incorporated a new paragraph summarizing this response on pages 7 and 8 of our manuscript.

We value the reviewer's consideration regarding the impact of the DNA arms in our constructs on the observed disassembly pathway using AFM. It is indeed heartening to note that our results align closely with the findings of numerous other studies addressing this pivotal subject. Initially, this was quite surprising to us, as we had anticipated more discrepancies, given that these prior studies were conducted under non-physiological conditions. To the best of our knowledge, this study marks the first instance of elucidating the nucleosome disassembly pathway by observing simultaneously both DNA and NCP, in real-time under physiological conditions. The fact that our results not only concur with previous research but also provide valuable supporting insights suggests that the extended DNA arms in our constructs did not have an adverse impact on the process. Moreover, it's worth highlighting that nucleosomes situated at terminal positions of chromatin arrays often feature free DNA ends. We explicitly acknowledge in our manuscript that our findings are most relevant to such scenarios (see page 4, lines 31-32).

3) The authors mention that DNA length could not be accurately measured due to high mobility (Pg.5, line 10). How to then assess the accuracy of the length measurements provided?

We apologize for the confusion regarding the measurement of DNA lengths in our HS-AFM experiments. As explained above in the answer to comment 1, the dynamics of the DNA arms are very complex, unpredictable, and convoluted with other dynamics. Moreover, because the surface was unmodified, often the DNA was partially or totally invisible due to their transient partial or total desorption from the surface. Thus, it was virtually impossible to measure frame-by-frame with accuracy the full length of each DNA arm. However, there were instances where both DNA arms were fully visible, this phenomenon can be observed in the provided SI movies. Therefore, we did find frames where both DNA arms were fully visible on the surface, we used only those frames to measure the DNA arm’s length. We have clarified this important issue in our manuscript (Pg., 4 lines 35-35 and pg.22 lines 580-584).

Minor concerns:

1) Pg.6, line 57: "Tracing of the short arm (flanking the 601 NPS rigid arm) and the long arm (flanking the 601 NPS flexible arm) are displayed as orange and green lines, respectively." Isn't the short arm green and long arm orange?

Indeed, the short arm is green colored while the long arm orange colored. We appreciate the observation very much; it has been corrected accordingly.

2) In Fig. 1B bottom panel, the angle of exit (theta 1) seems very fluctuating. There is a large change in angle at 10s time point which is not accompanied by any disassembly event. How relevant is it then to correlate disassembly events with angular changes?

We appreciate the reviewer's insights, which prompted a thorough reevaluation of our data and measurement algorithm. This led to significant improvements and additional results.
This question is related to a previous one in comment #1, we have partially addressed it in the previous response. By examining a broader set of molecules near the dimer ejection event, we have gained a nuanced understanding of angular changes across different nucleosomes. These variations are tied to nucleosome orientations, conformational states, and DNA wrapping. Notably, angular changes related to the first dimer ejection consistently occur at the exit site, while those associated with the second dimer's dissociation are observed at the entry site of the NCP. This information is visually presented in Figure SI 4 and detailed on page 7, lines 119-130.

Reviewer: 4

In this manuscript, the authors reported the real-time visualization of multistep asymmetrical disassembly of nucleosomes and chromosomes using High-Speed Atomic Force Spectroscopy. More specifically the authors have trained a neural network and developed an automatic algorithm that can track the structural changes in real time. This research could be valuable in understanding the nucleosome disassembly thereby studying the eukaryotic genome regulation. The manuscript is well written and would be interesting to the DNA and Chromosomal community. However, there are some concerns/comments the authors need to address/revise the manuscript can be accepted after incorporating them.

1. The authors used neural networks to delineate the various structural changes using AFM but the data is not provided regarding the output. The authors have mentioned ~95% prediction accuracy by training but there is no True Positive / False Positive and so on. The authors should provide a more detailed analysis of their neural network model i.e. Confusion matrix. Detailed information about machine learning is also missing. Is it supervised/unsupervised? I encourage the authors to brief about their prediction model in the main text as it is key in the prediction of dissembled structures.

We are grateful for the reviewer's valuable feedback and the request for further elaboration on the development of our new algorithm for dynamic AFM data analysis. Consequently, we have extended the content within the Data Analysis section of the Methods. This expansion includes more in-depth technical insights and a discussion of the limitations pertaining to the algorithm's development and performance, found on pages 21-23. To enhance clarity, we have also introduced a visual representation of the workflow employed for our machine learning implementation and a summary table presenting the algorithm's performance validation results (Fig. SI 2B and Table SI 2) as supporting materials to complement the method description.

2. The authors should also consider citing the important article from Kurumizaka-group http://dx.doi.org/10.1016/j.ymeth.2014.08.019 where they evaluated the nucleosome stability using

thermal stability assay. The authors should also correlate their AFM observations with earlier published results briefly in the manuscript.

We apologize for the oversight of this very relevant contribution available in the literature and thank the reviewer for bringing it to our attention. The report has been thoughtfully included as reference #24 and duly cited on page 2, as one of the numerous endeavors to comprehend the dynamics and stability of nucleosomes.
We address the comparison of our results with those reported by other authors on page 17 (lines 400 to 424).

3. I am also curious about the intermediate structures the authors mentioned, Can the authors explain in brief about the possibilities?

We appreciate the reviewer's interest in the intermediate conformations adopted by the molecules during our observations. As indicated throughout the manuscript (Pg., 6, line 81; pg. 9, line 150; pg. 14, lines 311 and 318; pg. 16, lines 358 and 364; and pg. 17, line 418-422), we have successfully captured reversible conformational transitions encompassing shifts from spherical to lobe or teardrop, as well as oblong morphologies of the NCP, alongside irreversible histone ejection events. However, we have maintained a conservative approach in discussing these findings due to the complexity of tip-force effects and limited AFM spatial resolution. Nonetheless, we suspect that these transitions are intricately linked to the nucleosomes' inherent plasticity, as proposed in the text (Page 16, line 364).
